# Arrhythmogenic Mechanisms in Heart Failure: Linking β-Adrenergic Stimulation, Stretch, and Calcium

**DOI:** 10.3389/fphys.2018.01453

**Published:** 2018-10-16

**Authors:** Daniel M. Johnson, Gudrun Antoons

**Affiliations:** ^1^Department of Cardiothoracic Surgery, Cardiovascular Research Institute Maastricht, Maastricht University, Maastricht, Netherlands; ^2^Department of Physiology, Cardiovascular Research Institute Maastricht, Maastricht University, Maastricht, Netherlands

**Keywords:** heart failure, myocytes, calcium, sympathetic stimulation, stretch, ryanodine, microdomains

## Abstract

Heart failure (HF) is associated with elevated sympathetic tone and mechanical load. Both systems activate signaling transduction pathways that increase cardiac output, but eventually become part of the disease process itself leading to further worsening of cardiac function. These alterations can adversely contribute to electrical instability, at least in part due to the modulation of Ca^2+^ handling at the level of the single cardiac myocyte. The major aim of this review is to provide a definitive overview of the links and cross talk between β-adrenergic stimulation, mechanical load, and arrhythmogenesis in the setting of HF. We will initially review the role of Ca^2+^ in the induction of both early and delayed afterdepolarizations, the role that β-adrenergic stimulation plays in the initiation of these and how the propensity for these may be altered in HF. We will then go onto reviewing the current data with regards to the link between mechanical load and afterdepolarizations, the associated mechano-sensitivity of the ryanodine receptor and other stretch activated channels that may be associated with HF-associated arrhythmias. Furthermore, we will discuss how alterations in local Ca^2+^ microdomains during the remodeling process associated the HF may contribute to the increased disposition for β-adrenergic or stretch induced arrhythmogenic triggers. Finally, the potential mechanisms linking β-adrenergic stimulation and mechanical stretch will be clarified, with the aim of finding common modalities of arrhythmogenesis that could be targeted by novel therapeutic agents in the setting of HF.

## Introduction

Heart failure is a complex clinical syndrome with many contributory factors including ischemia, congenital heart disease, and pulmonary hypertension. HF can be defined as HF with preserved ejection fraction (HFpEF), when diastolic dysfunction plays a major role, or HF with reduced ejection fraction (HFrEF). HF with reduced ejection fraction has been associated with elevated sympathetic tone and mechanical load (Lohse et al., 2003). Both systems activate signaling transduction pathways that increase cardiac output, but adversely contribute to electrical instability, at least partially via modulation of Ca^2+^ handling.

The first documentation of alterations in the sympathetic signaling in chronic HF was when a decrease in concentrations of the sympathetic nervous neurotransmitter, norepinephrine, was shown in the failing human heart (Chidsey et al., 1963). Since that time, there has been accumulating evidence that the sympathetic nervous system plays a considerable role in HF (Port and Bristow, 2001) and this is highlighted by the continued use of β-receptor blockers as a favorable pharmacological treatment of HF (Waagstein et al., 1993; Ponikowski et al., 2016).

The hyperadrenergic state is in large part caused by an imbalance of autonomic reflex responses to early alterations in cardiac and peripheral hemodynamics (Toschi-Dias et al., 2017). In HF, vagal control by the baroreceptor reflex is reduced (Eckberg et al., 1971), while sympatho-excitatory reflexes are augmented, including the cardiac sympathetic afferent reflex (Wang and Zucker, 1996). The cardiac-specific reflex originates in the ventricle and is activated by elevated filling pressures (Malliani et al., 1973; Wang and Zucker, 1996), creating a positive feedback loop as its activation causes excessive sympathetic outflow to the heart and arteries (Chen et al., 2015). In turn, the heart readapts its systolic and diastolic force to the adrenergic-mediated increases in hemodynamic load via intrinsic autoregulatory mechanisms (Neves et al., 2015). Thus, adrenergic and hemodynamic regulatory systems tightly interact via a complex interplay of feedback mechanisms at the local and systemic level that are initially compensatory, but ultimately pathological.

Arrhythmias are a major cause of mortality in HF patients, and sudden cardiac death has previously been linked with a higher NYHA class (Saxon et al., 2006; Santangeli et al., 2017). Furthermore, in a recent study, ventricular arrhythmias were seen in up to 45% of patients who had received a LVAD (Garan et al., 2013). Although over the last decades remarkable advances have been made in terms of our understanding of risk factors and the efficacy of device therapy the underlying mechanisms responsible for arrhythmia induction and sudden cardiac death in this population remain elusive, and this is largely down to the complexity of the disease.

In this review, we will focus on the roles that altered sympathetic stimulation as well as mechanics may have on arrhythmogenic phenotype in patients with HF with reduced ejection fraction, concentrating on alterations of Ca^2+^ dynamics, β-adrenergic stimulation and stretch at the level of the single cardiac myocyte. It is hoped that information gained in this field will ultimately lead to novel strategies that could improve our therapeutic arsenal against HF.

## Basic Principles of Calcium-Dependent Arrhythmogenesis-Afterdepolarizations

Before discussing arrhythmogenic mechanisms in HF we need to understand the basic mechanisms of arrhythmogenesis and the link to Ca^2+^.

**FIGURE 1:**
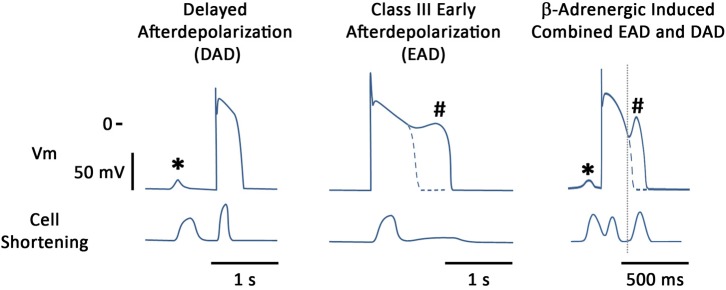
Stylized examples of afterdepolarizations occurring in the single canine myocyte. Figure shows both membrane potentials and contraction for each situation. **(Left)** DAD (^∗^) induced by the β-adrenergic agonist isoproterenol (ISO); **(Middle)** illustrates an EAD (#) induced by augmentation of the late sodium current, using ATX-II; **(Right)** illustrates that under certain conditions both types of afterdepolarizations can be seen in the same action potential. In this particular example, blockade of the potassium current, I_Ks_, together with β-adrenergic stimulation are the proarrhythmic treatment and it can be seen that an early aftercontraction initiates prior to the upstroke of the EAD.

Afterdepolarizations are thought to be one of the major mechanisms driving arrhythmogenesis in multiple patho-physiologies and we will concentrate on these in this review (**Figure 1**). These oscillations in the membrane potential can lead to either triggered activity and/or functional block which may encourage re-entry circuits (Wit and Rosen, 1983). These phenomena can be detected at multiple levels, ranging from the single cardiac myocyte to the tissue and can even be observed in the intact heart when monophasic APs are recorded (Priori et al., 1990). They are defined as depolarizations of the cardiac AP that can occur in phases 2, 3, or 4 of the AP (Cranefield, 1977). When they occur in phase 4 of the AP they are called DADs whereas if they occur earlier on the AP then they are termed EADs.

There is now a general consensus that DADs are a result of a I_TI_ activated by intracellular Ca^2+^ (Marban et al., 1986). This I_TI_ was originally described by Lederer and Tsien (1976) as a result of digitalis-induced arrhythmias and was later shown to be mainly due to activation of the electrogenic Na^+^-Ca^2+^ exchanger (NCX), while the Ca^2+^-activated Cl^-^ current (ICl_Ca_) appears to contribute in some species (Fedida et al., 1987; Zygmunt et al., 1998). Interestingly a study from Verkerk et al. (2001) showed that I_TI_ in failing human cardiac myocytes was as a result of NCX alone. In addition to the potential of DADs to induce arrhythmias via triggered activity or functional block, recent work has also highlighted the potential of DADs to increase beat-to-beat variability of repolarization (BVR) which may also contribute to the arrhythmogenic nature of these afterdepolarizations (Johnson et al., 2013). The Ca^2+^ that activates these arrhythmogenic currents originate from the SR and is released via diastolic release events (Venetucci et al., 2008). For these reasons, when the SR is overloaded with Ca^2+^, for example during intense β-adrenergic stimulation, the chance of spontaneous Ca^2+^ release is increased as is the likelihood for DADs (Yamada and Corr, 1992).

Mechanisms underlying EADs are much less clear cut however, and remain a topic of much debate. Early evidence suggested EADs were caused as a result of reactivation of I_CaL_ due to the prolonged plateau phase of the AP (January and Riddle, 1989; Zeng and Rudy, 1995). However, there is other experimental evidence that suggests that EADs may also be caused as a result of I_TI_ activation by intracellular Ca^2+,^ especially under conditions of Ca^2+^ overload (Priori et al., 1990; Volders et al., 1997, 2000). Either way, it appears that EADs are modulated by systolic release of Ca^2+^ during the AP and are regulated by feedback on Ca^2+^ sensitive ion currents.

The link of afterdepolarizations at the single myocyte level to arrhythmogenesis at the whole heart level is extremely complex and incompletely understood. In the intact heart, myocytes are electrically coupled to each other, meaning that the membrane voltage is governed by not just one cell but multiple cells. Furthermore, the contribution of one afterdepolarization occurring in one cell (the source) will likely be negated by the neighboring cells not experiencing afterdepolarizations (the sink). Elegant work from the laboratory of James Weiss has investigated this phenomenon and has shown that chaotic EADs are able to synchronize globally when the tissue is smaller than a critical size. However, when the tissue is large enough, complete synchronization of EADs can no longer occur and this results in regions of partial synchronization that shift in time and space, that can act as foci for arrhythmia (Sato et al., 2009). Furthermore, work from the same group also estimated that the number of local myocyte DADs needed to be synchronized to induce a premature beat would be very large, however, this could be reduced structural and electrical remodeling (Xie et al., 2010). Finally, a recent study from the Bers’ lab has also highlighted that in HF there is a much higher density of ‘Ca^2+^ asynchronous’ myocytes that are poorly coupled to the surrounding myocardium. These poorly coupled myocytes may also contribute to initiating triggered activity (Lang et al., 2017).

Over recent years it has become apparent that Ca^2+^ control of repolarization, and therefore also of arrhythmogenesis is more of a local rather than a global phenomenon. Both functional and structural microdomains dictate local Ca^2+^ concentrations, gradients and effector proteins. As these domains appear to be affected in HF, especially when we consider β-adrenergic stimulation and stretch we must also consider how this local control occurs.

## Local Control of Ca^2+^ Release at the Dyad

In cardiac myocytes, Ca^2+^ is centrally involved in many processes including excitability, contraction, and regulation of gene expression. Such diversity of functional roles postulates the existence of dedicated microdomains in which Ca^2+^ signals are generated independently of cytosolic Ca^2+^ concentrations and sensed by macromolecular signaling components localized to these microdomains. Besides a functional component, Ca^2+^ microdomains are often physically delimited by specialized membrane structures and subcellular compartments. Specialized structures include dyadic junctions between transverse membrane invaginations (T-tubules) and SR, sarcolemmal domains outside dyads such as lipid rafts and caveolae, and intracellular structures such as tethered junctions between SR and mitochondria. Interestingly, compartmentalization of proteins that generate or regulate microdomain Ca^2+^ signaling is dynamic, often as a cause or consequence of disease. For example, the LTCC, or nNOS coupled to RyR, can translocate from the dyad to the sarcolemma which alters their function, presumably by coupling to different signaling complexes (Sanchez-Alonso et al., 2016; Carnicer et al., 2017).

### Structural Organization

In the dyad, RyR in the SR juxtapose LTCC along T-tubules at close distances (∼12 nm) (Forbes and Sperelakis, 1983). Individual dyads control the process of Ca^2+^ induced Ca^2+^ release, or CICR. The elementary event is a Ca^2+^ spark released from the SR by the opening of RyR in a single Ca^2+^ release unit (Cheng and Lederer, 2008). When an LTCC opens during an AP, the local Ca^2+^ concentration in the dyad raises much more than cytosolic Ca^2+^, from a diastolic level of 100 nM to more than 10 μM, sufficiently to activate RyR (Cannell and Kong, 2012). Not all RyR are localized at dyads; non-coupled RyR are activated through propagated Ca^2+^ release with a delay. Therefore, a large heterogeneity of the tubular system (e.g., due to T-tubule loss in HF) causes dyssynchrony of subcellular Ca^2+^ release during systole (Heinzel et al., 2011). During diastole, few spontaneous Ca^2+^ sparks occur due to the relatively low sensitivity of RyR to resting Ca^2+^ levels. A spontaneous release event is spatially confined, but when the Ca^2+^ sensitivity or the RyR increases through phosphorylation or oxidation, or when SR Ca^2+^ load is high, more spontaneous Ca^2+^ sparks summate in time and space into propagating Ca^2+^ waves.

The structural design of the dyad also provides an optimal setting for feedback mechanisms of SR Ca^2+^ release on Ca^2+^-regulated membrane currents. Negative feedback through Ca^2+^ release-dependent inactivation of LTCC serves as a mechanism to limit Ca^2+^ influx during the initial phase of the AP (Sham, 1997). As release-dependent inactivation is immediate, following the fast rise and decline of local Ca^2+^ near dyads, some of the LTCC may recover from inactivation within a single beat during the AP plateau (Acsai et al., 2011). The local feedback of Ca^2+^ on LTCC may contribute to the intrinsic BVR of the AP in normal physiology (Antoons et al., 2015). Interestingly, the same study did not find a major role for the NCX in BVR, although immunohistochemistry and functional studies have suggested colocalization of a fraction of NCX with LTCC (10–15% of total NCX) sensing local Ca^2+^ release in the dyadic subspace (Acsai et al., 2011; Scriven and Moore, 2013). In support of this notion, modulation of (dyadic) Ca^2+^ sparks by both reverse mode and forward mode NCX has been demonstrated (Neco et al., 2010; Biesmans et al., 2011).

To regulate CICR, the dyad harbors a repertoire of kinases and phosphatases that form macromolecular complexes with LTCC and RyR and regulate their levels of phosphorylation. PKA and CaMKII are key to the regulation of LTCC and RyR in the β-adrenergic and stretch response. PKA is targeted to LTCC and RyR via AKAPs, and transmits signals from β-ARs via cAMP (Catterall, 2015; Landstrom et al., 2017). Dyadic cAMP signals in the vicinity of LTCC and RyR are controlled by localized PDE activity (Kokkonen and Kass, 2017). It should be noted that exact mechanisms behind PKA regulation of RyR and its specific role in the β-adrenergic response are incompletely understood. Marx et al. (2000) proposed that PKA phosphorylation dissociates FKBP12.6 from RyR thereby enhancing RyR open probability. However, this mechanism remains questionable (Xiao et al., 2004). CaMKII is dually activated by Ca^2+^ and ROS (Maier and Bers, 2007), and possibly also by NO at high Ca^2+^ levels during β-adrenergic stimulation (Curran et al., 2014). Although CaMKII is targeted to both coupled and non-coupled RyR via unknown mechanisms, its activation is confined to the dyad, where it enhances the open probability of RyR and LTCC (Wehrens et al., 2004; Bers and Morotti, 2014).

### Reactive Oxygen Species

In addition to phosphorylation mechanisms, ROS and NO have emerged as critical regulators of CICR. They modify LTCC and RyR function through redox modification of free cysteine residues. The action of ROS and NO is often multiphasic and bidirectional, depending on source, oxidant species, amount and timing and importantly, the local redox environment (Zima and Blatter, 2006). Typically, free radicals are short-lived and can only act on effectors in the close vicinity. Thus, redox modulation of Ca^2+^ in a cardiac myocyte is basically a tale of microdomain signaling of which the specific effects are determined by the subcellular location of the ROS/NO source and co-localization with its target proteins. Endogenous ROS are generated in the mitochondria as a by-product of respiration, and locally in the cytosol by specialized enzymes, such as NADPH oxidases (Burgoyne et al., 2012). Much of the O_2_^-^ produced is rapidly converted to H_2_O_2_, a more stable and membrane permeable derivative. Endogenous NO is produced in relatively low concentrations by endothelial and neuronal isoforms of NOS (eNOS and nNOS, respectively) (Massion et al., 2003).

An important player in the redox control of dyadic Ca^2+^ is NOX2, a membrane-bound NADPH oxidase that resides in T-tubules. NOX2 is induced by fast pacing and stretch, and activates RyR via *S*-glutathionylation (Sánchez et al., 2005). RyR activation by rapid pacing also requires CaMKII, which itself is redox regulated (Erickson et al., 2008). Interestingly, the NOX2-CaMKII regulation of RyR is restricted to the dyadic cleft. In pig myocytes that resemble human and have a significant population of non-coupled RyR, faster pacing significantly increased Ca^2+^ spark activity of dyadic RyR, but not the activity of non-coupled RyR. Additionally, NOX2 and CaMKII inhibition abolished Ca^2+^ sparks in dyadic regions, but not near non-coupled regions (Dries et al., 2013). At this point it cannot be concluded if NOX2-derived ROS is upstream of CaMKII oxidation [as suggested in models of oxidative stress induced by angiotensin (Erickson et al., 2008; Purohit et al., 2013)] or whether NOX2 and CaMKII act in parallel. Furthermore, exact mechanisms of microdomain-specific activation of NOX2 and CaMKII in response to rapid pacing remain elusive. NOX2 is also activated by stretch. Prosser et al. (2011) have demonstrated that stretching a myocyte triggered an immediate burst of ROS and Ca^2+^ sparks. The ROS was derived from NOX2 as the response was sensitive to NOX2 inhibitors and absent in NOX2 deficient mice (Prosser et al., 2011).

In contrast to NOX2, mitochondria constitutively produce ROS. Mitochondria are located at a very short distance of dyadic regions [between 37 and 270 nm based on electron microscopy analysis of rat myocardium (Sharma et al., 2000)]. Several studies have shown that mitochondrial ROS can activate RyR (reviewed in Zhang H. et al., 2013), suggesting that basal ROS production by mitochondria is responsible for a significant portion of spontaneous Ca^2+^ sparks (Yan et al., 2008).

The LTCC also acts as a redox sensor due to free thiol groups in its α1-subunit (Muralidharan et al., 2016). Reported ROS effects on Ca^2+^ channel function are both stimulatory (Song et al., 2010), or inhibitory (Gill et al., 1995). This discrepancy might be due to differences in the phosphorylation state of the Ca^2+^ channel. Several serine/threonine kinases that regulate the channel are subjected to ROS modification, including PKA, PKC, and CaMKII (see Burgoyne et al., 2012, for review). The positive effects of phosphorylation might partially counterbalance the inhibitory effects of direct ROS oxidation of LTCC. During high oxidative stress, LTCC facilitation by CaMKII is likely the predominant effect, since the calcium antagonist nifedipine could suppress the induction of EADs by H_2_O_2_ (Xie et al., 2009). ROS regulates many other proteins of the Ca^2+^ machinery outside the dyad. The overall effect of sustained ROS is Na^+^ and Ca^2+^ overload promoting even more ROS production via positive feedback and predisposing the cell to afterdepolarizations (Wagner et al., 2013).

Nitric oxide is both a positive and a negative regulator of excitation–contraction (EC) coupling underscoring the complexity of cardiac NO signaling (Simon et al., 2014; Farah et al., 2018). NO exerts its action via two pathways: an indirect pathway by the activation of sGC producing cGMP, and a direct pathway by *S*-nitrosylation of proteins. High levels of NO would predominantly stimulate the cGMP pathway causing negative inotropy, while low levels activate nitrosylation processes leading to positive inotropy (González et al., 2008). The mechanisms of nitrosylation, and particularly its effects on EC coupling, remain poorly understood. Despite much controversy, some consensus has emerged on the specific roles of eNOS and nNOS highlighting the importance of their subcellular localization in modulating Ca^2+^ handling proteins. Colocalization of eNOS and LTCC in caveolae at the sarcolemma favors *S*-nitrosylation and inhibition of LTCC (Wang et al., 2008). nNOS is targeted to the SR where it colocalizes with RyR and is therefore considered the prime NO modulator of dyadic Ca^2+^ (Barouch et al., 2002). NO nitrosylates RyR and increases its activity (Wang et al., 2010, but see also Zahradníková et al., 1997). The notion that the positive effects of nNOS are linked to its specific localization on the SR has been supported by a recent study that developed a transgenic mouse model in which nNOS was targeted to the sarcolemma and no longer co-localized with RyR (Carnicer et al., 2017). Interestingly, relocalization of nNOS, as may occur in HF, produced the same negative effects on I_CaL_ and contraction as eNOS. In normal physiology, NOS activity is controlled by β-adrenergic stimulation and stretch, as will be discussed in the next paragraphs.

## β-Adrenergic Signaling and Afterdepolarizations

In the heart, enhanced sympathetic activity is a potent stimulus for generation of arrhythmias. The relationship between sympathetic stimulation and triggered activity has long been recognized, *in vivo* (Priori et al., 1988, 1990) and *in vitro* (Lazzara and Marchi, 1989). During β-adrenergic stimulation, DADs and EADs often coexist. When Ca^2+^ overload plays a role in afterdepolarization formation, as could be the case under β-adrenergic stimulation, both EADs and DADs can be abolished by ryanodine, suggesting a common dependence of these on SR Ca^2+^ release under these conditions. In cardiac myocytes, β-ARs and their effector pathways targeting Ca^2+^ handling proteins are highly compartmentalized. In this paragraph, we will discuss the parallel activation of multiple molecular pathways by β-adrenergic subtypes, their specific end targets to controlling local and global Ca^2+^ release, and their impact on the generation of DADs and EADs.

### β-Adrenergic Signaling Pathway

**FIGURE 2:**
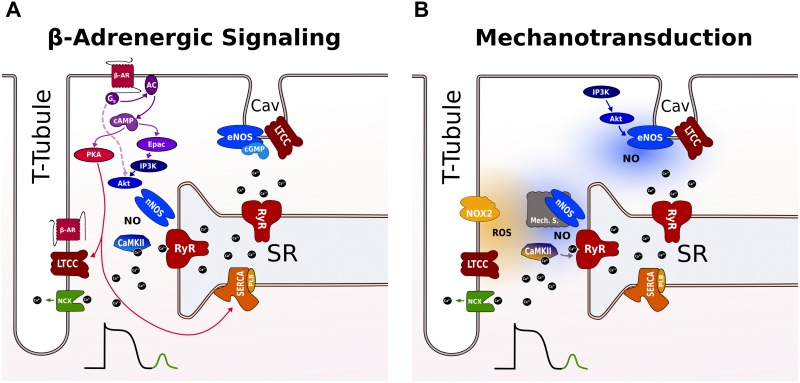
Proposed pathways for inducing SR Ca^2+^ leak during β-adrenergic signaling and stretch in a ventricular cardiomyocyte. **(A)** β-AR raises cAMP levels via G_s_-protein-dependent activation of AC that activates both PKA and Epac. PKA phosphorylates LTCC and PLB leading to more Ca^2+^ influx and faster uptake by SERCA into the SR. Epac activates nNOS and CaMKII via an PI3K and AkT signaling cascade promoting SR Ca^2+^ leak via RyR phosphorylation. The broken line indicates a cAMP and Epac-independent pathway for local activation of nNOS targeted to RyR in the dyad. RyR not coupled to LTCC in T-tubules are not modulated by CaMKII and nNOS. eNOS is localized to caveolae and exerts negative effects on LTCC during β-adrenergic stimulation. **(B)** Mechanotransduction involves ROS and NO for RyR activation. The ROS and NO pathway are independent and operate on different timescales via different mechanosensors. NOX2 produces ROS near RyR in the dyad increasing RyR activity possibly via oxidation of CaMKII. With a delay, nNOS is activated via an unknown mechanosensing mechanism. The enhanced SR Ca^2+^ leak promotes Ca^2+^ waves that activate a transient inward NCX current causing DAD. Caveolar eNOS is activated by stretch via PI3K-Akt and positively modulates EC coupling outside the dyad by mechanisms that are incompletely understood. See text for further details. AC, adenyl cyclase; β-AR, β-adrenergic receptor; cAMP, cyclic adenosine 3’,5’-monophosphate; CaMKII, Ca^2+^/calmodulin-dependent protein kinase II; EC, excitation contraction; DAD, delayed afterdepolarizations; eNOS, endothelial nitric oxide synthase; Epac, exchange protein activated by cAMP; LTCC, L-type Ca channel; NCX, Na^+^/Ca^2+^ exchanger; NO, nitric oxide; NOX2, NADPH oxidase type 2; nNOS, neuronal nitric oxide synthase; PI3K, phosphoinositide 3-kinase; PKA, protein kinase A; PLB, phospholamban; ROS, reactive oxygen species; RyR, ryanodine receptor; SERCA, SR Ca^2+^-ATPase; SR, sarcoplasmic reticulum.

β-Adrenergic stimulation activates two pathways that operate in parallel: a PKA-dependent pathway that impacts on systolic Ca^2+^ through modulation of SR Ca^2+^ load, and a CaMKII pathway that regulates diastolic SR Ca^2+^ release (**Figure 2A**). Molecularly, the PKA signaling cascade is clearly defined. Upon activation, β-adrenergic agonists stimulate adenylate cyclase via Gs-coupled proteins raising cAMP levels that activates PKA (Bers, 2002). Subsequent phosphorylation of PKA substrates, including LTCC (causing increased Ca^2+^ influx) and PLB (accelerating SR Ca^2+^ uptake), results in enhancement of SR Ca^2+^ load. In cardiac myocytes, the compartmentation of cAMP signaling has been attributed to different β-AR subtypes that have distinct subcellular locations. β2 receptors are preferentially located at T-tubules where they co-localize with LTCC in caveolae, while β1 receptors are distributed more globally across T-tubules and surface sarcolemma. Using FRET sensors for cAMP, it was demonstrated that selective β1 stimulation generates cAMP signals that propagate throughout the cell, whereas the β2 AR signal is locally confined in T-tubules and specifically regulates LTCC during CICR (Nikolaev et al., 2006).

*In vitro*, PKA can also phosphorylate RyR (Marx et al., 2000), but in intact myocytes a functional role for PKA regulation of RyR remains controversial. Most evidence is in favor of CaMKII as the prime modulator of RyR during β-adrenergic stimulation. In response to adrenergic activation, CaMKII phosphorylates RyR and increases open probability when measured as SR Ca^2+^ leak (Curran et al., 2007) or diastolic Ca^2+^ sparks (Gutierrez et al., 2013). A recent study in pig myocytes suggested that CaMKII-dependent modulation of RyR during β-adrenergic stimulation was restricted to RyRs specifically in the dyadic cleft, and involved local activation of nNOS (Dries et al., 2016). The nNOS/CaMKII effects were not observed in RyR release sites that were not coupled to T-tubules. While there is conclusive evidence to support nNOS involvement in β-adrenergic modulation of RyR (Massion et al., 2003), the steps upstream from nNOS activation are less well defined. Some, but not all, studies suggested the involvement of Epac, a cAMP target parallel to PKA, leading to CaMKII autophosphorylation and downstream RyR phosphorylation (Pereira et al., 2007; Oestreich et al., 2009). A second pathway, independent of cAMP, involves PI3K and Akt as upstream activators of nNOS inducing CaMKII activation via nitrosylation (Curran et al., 2014). A recent study suggested that the Epac and nNOS pathway are interdependent and function largely in series (Pereira et al., 2017).

During β-adrenergic stimulation, the cAMP-PKA and nNOS-CaMKII pathways operate in parallel. From the current evidence it is clear that PKA signaling is distributed more globally with robust effects on LTCC and SERCA, whereas CaMKII is highly localized and exerts stronger effects than PKA on RyR. Ca^2+^ current facilitation by CaMKII under β-AR has also been demonstrated (Dries et al., 2016). The integrated response is enhancement of LTCC currents, SR Ca^2+^ load and leak. Together, these effects are able to favor both EADs and DADs.

### Ca^2+^-Dependent Mechanisms of Afterdepolarizations During β-Adrenergic Stimulation

The link between β-adrenergic stimulation, RyR-mediated Ca^2+^ leak and arrhythmogenesis has been most convincingly demonstrated in the clinical case of catecholaminergic polymorphic ventricular tachycardia (CPVT). Patients with CPVT carry mutations in RyR that increase the open probability of the receptor, or in calsequestrin (Casq2) where SR Ca^2+^ buffering is hindered and/or the interaction of Casq2 and RyR is altered (Cerrone et al., 2009). Introducing a CPVT associated RyR mutation in a mouse model, for example, resulted in a higher rate of Ca^2+^ sparks, waves and DADs in myocytes, and development of bidirectional ventricular tachycardia after exposure to catecholamines (Cerrone et al., 2005; Liu et al., 2006; Fernández-Velasco et al., 2009). The higher incidence of DADs and triggered activity has been attributed to increased RyR sensitization to Ca^2+^ (lowering the SR threshold for Ca^2+^ waves), and the enhanced SR Ca^2+^ by catecholamines (Fernández-Velasco et al., 2009; Kashimura et al., 2010). DAD occurrence also critically depends on the balance between SR Ca^2+^ load and the diastolic interval. Short durations reduce the time for SR refilling and recovery, and hence the likelihood of DADs. CPVT patients sometimes develop bradycardia. In this population the slow supraventricular rate has been suggested as the primary cause of ventricular arrhythmias (Faggioni et al., 2013), which may explain the reduced response of some patients to β-blockers (Priori et al., 2002). During bradycardia, particularly the Purkinje cells of the conduction system are prone to developing DADs and present a major source of focal activity in CPVT (Cerrone et al., 2007).

In conditions of excessive Ca^2+^ load, DADs and EADs often appear simultaneously (Priori and Corr, 1990; Volders et al., 1997; Antoons et al., 2007). A study by Johnson et al. (2013) in dog ventricular myocytes proposed a mechanism that coupled diastolic DADs to increased BVR and EADs. In the presence of isoproterenol, diastolic Ca^2+^ waves and DADs frequently appeared between beats. After a DAD, the duration of the next AP was consistently prolonged, related to an increase in I_CaL_. The Ca transient during CICR was smaller after a DAD (presumably due to partial SR depletion), and modeling and voltage-clamp analysis explained the I_CaL_ facilitation by a reduction of Ca^2+^ dependent inactivation of I_CaL_. The feedback of DAD on I_CaL_ caused significant BVR. Furthermore, because of the prolonged AP after a DAD, new Ca^2+^ waves could be generated before the end of repolarization and form EADs.

The phenomenal observation of Ca^2+^ sparks and waves that occur due to spontaneous (not triggered) openings of RyR clusters during diastole is well known (Cheng and Lederer, 2008). Triggered Ca^2+^ sparks, i.e., produced by LTCC openings during CICR, occur near-synchronously at the start of the Ca^2+^ transient and are not expected to occur during relaxation because of refractoriness of the RyR and SR Ca^2+^ content. However, using high sensitivity detectors during confocal imaging in rabbit ventricular myocytes, Fowler et al. (2018) detected Ca^2+^ sparks during the decay of the Ca^2+^ transient. They explained the occurrence of these late Ca^2+^ sparks by the ability of release sites to recover from refractoriness during the plateau phase of the AP to become reactivated either by cytosolic Ca^2+^ itself, or by stochastic openings of LTCC. Late Ca^2+^ sparks are more readily observed when CaMKII activity is increased, as was reported in a mouse model of CaMKIIδc overexpression (Guo et al., 2012). CaMKII phosphorylation of LTCC causes a shift in the distribution of LTCC into high-activity gating modes and accelerates recovery from inactivation (Sham et al., 1995; Guo and Duff, 2006), which could explain the facilitation of late Ca^2+^ sparks by CaMKII. Interestingly, repetitively firing of late Ca^2+^ sparks produced microscopic waves of Ca^2+^ release presenting a new paradigm of electrical instability underlying BVR and EAD (induced by a DAD-like mechanism during the AP plateau), particularly in settings of HF with prolonged AP and increased CaMKII activity.

A final mechanism of EAD seen with β-AR relates to the dynamic modulation of Ca^2+^ window currents through Ca^2+^-dependent feedback. The mechanism is independent of spontaneous Ca^2+^ events (unlike the mechanisms discussed above) but reflects local feedback of SR Ca^2+^ release on LTCC during CICR. In the classical view, EAD are caused by voltage-dependent recovery of inactivated LTCC. Inactivation and recovery of LTCC also have a Ca^2+^-dependent component (Sipido et al., 1995; Sham, 1997), dynamically shaping Ca^2+^ window currents during a single beat. In dog and pig myocytes, we observed that under isoproterenol Ca^2+^-dependent recovery of window currents was faster than the decay of global Ca^2+^ transients, relatively unaffected by slow Ca^2+^ buffers and absent when SR Ca^2+^ release was inhibited (Antoons et al., 2007; Acsai et al., 2011). These data strongly suggest that release-dependent recovery of window currents is driven by local changes in dyadic Ca^2+^. The Ca^2+^-dependent regulation of Ca^2+^ window currents seem to require sufficiently high levels of dyadic Ca^2+^, or local activation of CaMKII, as the dynamic inactivation and recovery process was no longer observed in the absence of isoproterenol. Enhanced dynamic modulation of window LTCC by dyadic Ca^2+^ release is a suggested source of BVR (Antoons et al., 2015), and may contribute to an increased incidence of EADs under β-AR stimulation (Antoons et al., 2007).

## Mechanical Load and Afterdepolarizations

Acute stretching of the heart destabilizes membrane potential and causes DADs and EADs (Franz et al., 1989). This arrhythmogenic activity is caused by negative feedback mechanisms that integrate mechanical and electrical activity of cardiac myocytes and presumably involve Ca^2+^ (Ravens, 2003). The myocardium responds to stretch by a more powerful contraction, a phenomenon referred to by Frank Starling (Sagawa et al., 1990). The intrinsic adaptation to changes in mechanical load has a second slower component of enhanced contractility described by the Anrep effect (von Anrep, 1912). Early work in intact cardiac muscle had not observed significant changes in diastolic and systolic global Ca^2+^ levels during the initial stretch response (Allen and Kurihara, 1982), which has argued against a major role for Ca^2+^ in stretch-induced arrhythmias. More recently, this view has been challenged by experiments that confocally monitored Ca^2+^ sparks and waves during stretch, suggesting that local Ca^2+^ release could account for at least part of the Frank Starling response (Petroff et al., 2001). Since then, a complex picture of mechanosensitive Ca^2+^ signaling has emerged that operates over a wide range of time scales. Within milliseconds, a small diastolic stretch triggers a burst of Ca^2+^ sparks (Iribe et al., 2009). When sustained, Ca^2+^ accumulates over minutes via stretch-induced autocrine/paracrine signaling participating in the Anrep effect (Cingolani et al., 2013). When stress becomes chronic, elevated Ca^2+^ influx activates gene expression leading to hypertrophy (Tavi et al., 2001; Gómez et al., 2013).

### RyR Mechanosensitivity

Stretch-dependent regulation of the Ca^2+^ system is operated via the process of mechanotransduction. Its mechanisms involve many signaling cascades targeting a diversity of intracellular Ca^2+^ sources, including the SR and mitochondria (Schönleitner et al., 2017). Furthermore, mechanotransduction operates via different classes of mechanosensors of which the activation seems to depend on the mechanical environment of the myocyte, which in experimental settings is defined by the dimensionality of the stretch system (Chen-Izu and Izu, 2017). The modulation of RyR by mechanical force has been a focus of investigation after the first demonstration in a 3D cell-in-gel system that stretch can trigger Ca^2+^ sparks (Petroff et al., 2001). Subsequently, ROS and NO have been identified as key molecules in RyR mechanosensitivity (**Figure 2B**). NOX2 activation has been proposed as the principle mechanosensor underlying the initial fast response of Ca^2+^ sparks to stretch (Prosser et al., 2011). Stretch-induced ROS by NOX2 is fast, transient and confined near the dyad to permit rapid and reversible modification of RyR. It is therefore believed that the NOX2 pathway enhances CICR efficiency without changes in systolic Ca^2+^ and serves as an adaptation to beat-to-beat variations in preload contributing to the Frank Starling response.

Cardiac stretch also stimulates cardiomyocytes to produce NO (Khairallah et al., 2012). Mechanical stimulation of NO elevates the systolic Ca^2+^ transient and produces spontaneous Ca^2+^ sparks during diastole, as was demonstrated in myocytes contracting in-gel against a higher preload or afterload (Petroff et al., 2001; Jian et al., 2014). In these settings, NO was produced through activation of constitutive NOS by phosphorylation via the PI3K-Akt pathway (Petroff et al., 2001). Pharmacological inhibition or genetic deletion to differentiate between eNOS and nNOS pathways revealed that both isoforms were involved in the downstream effects on systolic Ca^2+^, but only nNOS had a role in the induction of Ca^2+^ sparks (Jian et al., 2014). The divergent effects of eNOS and nNOS have been explained by their subcellular location. nNOS is localized at the dyad in close proximity of RyR, while eNOS is spatially confined in caveolae more distant from RyR release sites (Xu et al., 1999). Downstream from nNOS signaling, CaMKII was also found to modulate afterload-induced Ca^2+^ sparks. The mechanically induced SR Ca^2+^ leak by nNOS is expected to deplete the SR of Ca^2+^, however, SR Ca^2+^ content is maintained presumably via enhanced SERCA Ca^2+^ reuptake by nNOS (Vielma et al., 2016).

The NO-mediated increase of the Ca^2+^ transient to compensate for greater mechanical load typically appears with a delay of seconds and minutes, possibly participating in the Anrep effect. Trans-sarcolemmal Ca^2+^ influx also contributes to the slow Ca^2+^ loading during stretch. One of the proposed mechanisms is increased activity of Na^+^/H^+^ exchanger through mitochondrial ROS release downstream of stretch-induced angiotensin signaling (Cingolani et al., 2013). The result is an increase in intracellular Na^+^ that stimulates reverse NCX loading the cell with Ca^2+^. Na^+^ and Ca^2+^ influx through non-selective cationic SAC may further contribute (Calaghan et al., 2004). Thus, slow adaptation to stretch is viewed as an enduring signal achieved by concerted action of local nNOS activity to fine-tune local Ca^2+^ release and transsarcolemmal Ca^2+^ and Na^+^ influx to gain more Ca^2+^.

### Ca^2+^-Dependent Mechanisms of Stretch-Induced Arrhythmias

While mechano-sensitization of RyR is part of an effective adaptation to preload and afterload by increasing the efficiency of local Ca^2+^ release, it also produces spontaneous Ca^2+^ sparks during diastole. In the normal heart, stretch-induced Ca^2+^ sparks are locally confined. Under certain conditions, when more Ca^2+^ sparks arise synchronously to form Ca^2+^ waves, the load-adaptive Ca^2+^ system could turn into an arrhythmogenic mechanism. The stretch-induced increase in ROS, Ca^2+^ sparks and velocity of propagating Ca^2+^ waves is graded, i.e., increases with increasing amount of stretch (Miura et al., 2015). Thus, large stretches, such as in dilated hearts, are more likely to trigger ventricular ectopy (Hansen et al., 1990). Mechanical dyssynchrony, often due to structural tissue heterogeneity, is a further compromising factor. In case of dyssynchronous contractions, Ca^2+^ waves arise from a non-SR source as result from Ca^2+^-dissociation from the contractile filaments during late relaxation of the non-uniform cardiac muscle (Miura et al., 2008).

More ROS can also by produced by hypersensitivity of mechanosensitive signaling due to upregulation of a molecular component, as was demonstrated for a mouse model of Duchenne muscular dystrophy that showed upregulation of NOX2 and produced Ca^2+^ waves in response to moderate stretch (Prosser et al., 2011). In addition to DADs, ROS also activates EADs, via reactivation of I_CaL_ (Song et al., 2010), or enhanced late Na^+^ current (Song Y. et al., 2006). While RyR, INaL, and LTCC can be directly activated by oxidation (Xu et al., 1998; Morita et al., 2003; Kassmann et al., 2008), redox modification of CaMKII seems to be crucially involved in ROS modulation of arrhythmogenic INaL and LTCC currents (Morita et al., 2009; Wagner et al., 2011). Of note, most electrophysiology studies applied H_2_O_2_ as an exogenous source of ROS. There are no current data to confirm if endogenous ROS produced by NOX2 during stretch behaves similarly. Source matters, as mitochondrial ROS caused a reduction of INa (Liu et al., 2010).

While ROS is a ubiquitous proarrhythmic signal, NO generate opposite pro- and antiarrhythmic signals that can be partly explained by divergent effects of eNOS and nNOS on Ca^2+^ handling proteins. Mice with targeted disruption of eNOS had a higher incidence of arrhythmias induced by ouabain (Rakhit et al., 2001) or β-adrenergic stimulation (Wang et al., 2008), confirming earlier work reporting on the protective effects of NO against ventricular arrhythmias in dogs (Vegh et al., 1992). The antiarrhythmic effects have been attributed to β-adrenergic antagonism of eNOS via reduction of I_CaL_ in a cGMP-dependent manner. Likewise, nNOS knockout mice suffered more from arrhythmias after myocardial infarction than their WT littermates. Because an I_CaL_ blocker reduced VF incidence, the authors concluded that nNOS is antiarrhythmic through I_CaL_ inhibition via direct nitrosylation (Burger et al., 2009). Nitrosylation of the Na^+^ channel is also coupled to nNOS activity (Ahern et al., 2000). Conversely, when nNOS is activated by stretch or catecholamines in cardiac myocytes, local NO-CaMKII signals produce arrhythmogenic Ca^2+^ waves that originate from dyadic RyR (Curran et al., 2014; Jian et al., 2014). Giving the pro-arrhythmic actions of isoproterenol *in vivo*, it is reasonable to argue that during β-adrenergic stimulation in the presence of mechanical load, pro-arrhythmogenic nNOS signaling prevails.

In unloaded myocytes, the I_TI_ following a Ca^2+^ wave is mainly produced by NCX. In stretched myocytes, a significant contribution of stretch-activated channels is anticipated. Stretch-activated non-selective cation currents (SACNS) have been functionally demonstrated in ventricular myocytes at the whole-cell and single-level (Craelius et al., 1988). While it is unlikely that Na^+^ and Ca^2+^ conducting SACNS participate in stretch-induced SR Ca^2+^ release in ventricular myocytes (Iribe et al., 2009), they may contribute to destabilize the resting membrane potential by generating inward current during diastole. Studies in whole hearts demonstrating anti-arrhythmic effects of GsMTx-4, a specific SACNS blocker, support the involvement of SAC in stretch-induced arrhythmias (Wang et al., 2016). The search for a ‘true’ SACNS, a structural homolog to the bacterial SAC that can be directly gated by membrane tension (Sukharev et al., 1994), is still ongoing. Interestingly in this regard is the discovery of Piezo channels in a neuroblastoma cell line (Coste et al., 2010). The biophysiological profile of Piezo matches endogenous cardiac SACNS, including weak voltage dependency, single channel conductance, inactivation, and sensitivity to GsMTx-4 (Gottlieb, 2017), and is therefore a promising candidate. Piezo is expressed at low levels in the heart (Coste et al., 2010), but its role in cardiac function has yet to be established.

In the heart, the search for cardiac SACNS has been largely focused on the transient receptor potential canonical (TRPC) channel family. The activation of TRP channels is polymodal, and some members are directly activated by membrane deformation (Inoue et al., 2009), although this remains somewhat controversial (Gottlieb et al., 2008). Two subtypes, TRPC3 and TRPC6, have been proposed as potential candidates participating in the slow force response (Yamaguchi et al., 2017). Hyperactive TRPC3 (Doleschal et al., 2015) or TRPC6 (Seo et al., 2014) amplified the slow inotropic response to stretch resulting in Ca^2+^ overload and arrhythmogenesis. Doleschal et al. (2015) explained the pro-arrhythmia of TRPC3 by a Ca^2+^ overload dependent mechanism that involves spatial uncoupling between TRPC3 and NCX1 in specialized microdomains disrupting the tight regulation of NCX by local Ca^2+^ and Na^+^. This thinking is in line with the conceptual view that TRPC channels have access to localized Ca^2+^ signaling microdomains that are separated from contractile dyadic signaling (Houser and Molkentin, 2008). The microdomain concept was initially proposed to explain the role of TRPC channels in the activation of the NFAT/calcineurin axis linking pathophysiological hypertrophy to chronic mechanical stretch (Kuwahara et al., 2006; Makarewich et al., 2014). It has been well established that structural and functional remodeling in pathological cardiovascular stress predisposes the heart to arrhythmias (Nattel et al., 2007; Orini et al., 2017).

### Linking Mechanotransduction and Adrenergic Signaling

Thus far, ROS, NO, and CaMKII have been identified as the prime mediators of RyR mechanosensitivity in the intrinsic adaptation of contractile force to load. *In vivo*, intrinsic force adaptation is modulated by sympathetic activation by imposing a higher load on the heart through modulation of vascular tone. The myocyte can respond to higher mechanical and adrenergic stress through activation of mechanotransduction and adrenergic signaling networks, as discussed above and depicted in **Figure 2**, but interactions have not been systematically investigated. The mechanotransduction pathway shows both the rapid preload-induced NOX2 and slower afterload-induced NO branch, that most probably operate independently (Jian et al., 2014). It is also unlikely that NOX2 is directly involved in β-adrenergic signaling, since ROS scavengers failed to prevent increases in Ca^2+^ spark frequency in quiescent cells that were treated with isoproterenol (Gutierrez et al., 2013). It should be noted that NOX2 can possibly become activated during β-adrenergic signaling as an indirect consequence of chronotropic effects (Dries et al., 2013).

nNOS is centrally involved in both stretch- and adrenergically induced Ca^2+^ sparks, most likely via oxidation of downstream CaMKII (Gutierrez et al., 2013; Jian et al., 2014). It is therefore tempting to speculate that nNOS and CaMKII act as primary integrators of mechanotransduction and adrenergic RyR signaling networks. The assumption that co-activation of nNOS has a cumulative effect on RyR activity remains to be determined.

The eNOS effects are less clearly defined. eNOS is compartmentalized in caveolae at T-tubules and sarcolemma. In sarcolemmal caveolae, eNOS colocalizes with β-ARs and LTCC allowing NO to mitigate β-adrenergic inotropy through inhibition of LTCC by local cGMP (Wang et al., 2008). It is conceivable that a stretch activation of the eNOS-Akt-PI3K pathway positively modulates EC coupling gain in T-tubular caveolae, while negatively regulating the β-adrenergic response in a different subset of caveolae at the surface sarcolemma.

## Heart Failure and Afterdepolarizations

Heart failure is associated with extensive cardiac remodeling, at both the structural and functional levels. Remodeling due to HF occurs for a number of reasons, however, it is in part, due to altered stress on the ventricular wall (Kehat and Molkentin, 2010).

Remodeling can lead to an increased propensity for complex ventricular arrhythmias and sudden cardiac death, and these are seen in over half of the patients presenting with HF with reduced ejection fraction. For these reasons it is imperative to understand the mechanisms that are responsible for the increased arrhythmia incidence in this population (Janse, 2004).

Purkinje fibers isolated from infarcted sections of human hearts have been shown to have significantly longer APD than those from non-infarcted zones, resulting in marked dispersion of APD in infarcted and adjacent zones. Furthermore, both epinephrine and the cardiac glycoside, ouabain, were able to induce DADs in these fibers (Dangman et al., 1982). Previous work using human trabeculae has also shown that there is an increased propensity for triggered activity in tissue from HF patients (Vermeulen et al., 1994). Further work from the Amsterdam group also showed that, in contrast to many animal species, norepinephrine induces APD prolongation in ventricular myocytes from human failing hearts, as well as EADs. These alterations were ascribed to an increase in both the calcium peak current and window current (Veldkamp et al., 2001).

In addition to the alterations in arrhythmia incidence in HF, it has been well described that the failing heart has a reduced responsiveness to elevated catecholamine levels, at least in end-stage HF, due to alterations in expression of β-ARs (Bristow et al., 1982; Ungerer et al., 1993). Interestingly, more recent work has also shown that in a patient cohort with HF, BVR of ventricular AP duration was increased during an autonomic challenge associated with increased sympathetic activity (Porter et al., 2017).

Taking these data together leads us to believe that modifications in signaling underlying β-adrenergic responsiveness and stretch may contribute to the increased occurrence of arrhythmias in these patient populations. Therefore, if we are able to understand the precise changes that occur in these systems during HF, we may get a better hold on the processed occurring, with an outlook of preventing and/or treating the, potentially, maladaptive remodeling (see below).

### Global Remodeling

At the gross structural level, the geometry of the heart changes as a result of HF, becoming less elliptical and more spherical (Cohn et al., 2000). HF is associated with a progressive enlargement of the left ventricle, with increases in end-systolic left ventricular wall stress being seen (Florea et al., 1999), which may have detrimental effects on mechanosensitive mechanisms involved in arrhythmia formation, and also contribute to the cellular arrhythmogenic remodeling.

#### Ion Channel Remodeling

At the level of the single myocyte, changes in HF include alterations in the densities of various membrane channels, which contributes to the increase in APD seen in the majority of HF models and in patients (Beuckelmann et al., 1993; Tomaselli and Marbán, 1999). One of the most consistent findings with regards to current alterations in HF is the decrease in the inwardly rectifying potassium current, I_K1,_ which contributes to maintaining the resting membrane potential as well as contributing to terminal repolarization (Beuckelmann et al., 1993; Nerbonne and Kass, 2005). Furthermore, the β-adrenergic regulation of I_K1_ has also been shown to be significantly reduced in myocytes isolated from HF patients (Koumi et al., 1995). Reduced I_K1_, will mean that a smaller I_TI_ will be required to cause the same amplitude of DAD, or even triggered AP, and therefore altered regulation of this current in HF has major implications in the potential arrhythmogenic outcomes. An interesting recent study, however, showed that sympathetically -induced arrhythmias could not be induced when I_K1_ was inhibited in isolation in Langendorff-perfused rabbit hearts indicating that synergistic activity between multiple pathways, including altered RyR sensitivity, was required for arrhythmia induction (Myles et al., 2015).

Another potassium current that is of great interest when it comes to β-adrenergic modulation, is the slow rectifier, I_Ks_. I_Ks_ function is prominent during β-adrenergic stimulation when it promotes AP shortening, to counteract the increase in inward Ca^2+^ current, thus providing critical “reserve” when other repolarizing currents are impaired (Jost et al., 2005; Varró et al., 2000; Volders et al., 2003). Although Veldkamp et al. (1995) could not detect this current in myocytes isolated from patients with cardiomyopathy, a number of animal models have indicated that this current is downregulated in HF (Tsuji et al., 2000; Li et al., 2002). A decrease in this current during intense sympathetic stimulation will lead to an increase in APD, and an increased tendency for afterdepolarizations. Furthermore, research from our own group has shown the key role that I_Ks_ plays in preventing excessive BVR during β-AR stimulation, which may also contribute to the arrhythmogenic substrate generated when this current is downregulated (Johnson et al., 2010, 2013).

The importance of both I_K1_ and I_Ks_ and their regulation by β-adrenergic stimulation in HF were recently highlighted by a study from the Bers’ group. In this manuscript, the physiologically relevant AP-clamp technique was utilized to show that under β-adrenergic stimulation, reduced I_Ks_ responsiveness limits the integrated repolarizing potassium currents in a rabbit model of HF. Furthermore, an increase in APD BVR was seen in HF myocytes. Taken together these data illustrate the importance that these currents may play in arrhythmia generation in HF, especially under sympathetic stimulation (Hegyi et al., 2018a).

Apart from the acute effects of adrenergic stimulation on channel activity, one must also consider the effect of sustained sympathetic activation. A recent study did just this by investigating the effects of sustained adrenergic stimulation on I_Ks_ dynamics. In that particular study, they showed that I_Ks_ was reduced after continued β-AR stimulation, and this was mediated by CaMKII, a signaling molecule involved in both β-AR and mechanosensitive stimulated arrhythmias (Shugg et al., 2018). If this effect contributes to the increased incidence of arrhythmias in HF is currently unknown and should be the subject of further work.

With regards to the acute effect of HF on SACNS, that may also contribute to stretch-induced arrhythmias, multiple laboratories have shown that TRPC channel expression and activity are upregulated in pathological hypertrophy and HF (Eder and Molkentin, 2011). Furthermore, to our knowledge, to date only one study has investigated the level of Piezo channels in HF, with that study providing evidence of an upregulation in HF. However, the functional consequences of this upregulation are currently unknown and should be the subject of further research (Liang et al., 2017).

#### Excitation–Contraction Coupling Remodeling

As previously stated, the synchronous rises in Ca^2+^ leading to efficient ECC is due, in part, to the tight opposition of RyRs and LTCCs in the T-tubules in healthy ventricular myocytes. There is an abundance of literature describing a loss of T-tubules during HF (Lyon et al., 2009; Guo et al., 2013; Dries et al., 2018a), In addition to the loss of the concerted effort for successful ECC that the loss of T-tubules will bring, deleterious Ca^2+^ handling leading to arrhythmia may also result. A recent study investigated the potential mechanisms behind T-tubule disruption in post-infarction failing rat hearts. In that study, they showed that elevated wall stress was associated with disruption of the T-tubular structure and this was associated with decreased levels of junctophilin 2, which is a critical dyadic anchor. Furthermore, they carried out studies on loaded papillary muscles, which confirmed a direct role of wall stress on regulation of T-tubule organization (Frisk et al., 2016). Taken together these data indicate the importance that stretch has in developing the HF phenotype when it comes to subcellular structure of the myocyte. Alterations in location of relevant signaling pathways that may also be induced by this loss of cellular architecture will be discussed later (see the Section “Local (Microdomain) Remodeling”).

As the current generated via the NCX appears to be the major player responsible for the I_TI_ that initiates DADs, and perhaps EADs, one also needs to consider how the function of this exchanger is altered in HF. Interestingly, a number of *in vitro* studies have suggested that stretch of adult myocytes increases NCX expression (Sipido et al., 2002). These data may lead us to believe that the increase stretch ‘felt’ by the *in situ* myocyte may also lead to an increase in NCX in HF. Indeed, the majority of studies have shown that NCX is increased in HF (Sipido et al., 2002; Schillinger et al., 2003), although we should approach these data with caution due to the fact that expression levels do not necessarily give an indication of activity, especially when considering an exchanger where ion concentrations, phosphorylation state [of partner proteins (e.g., phospholemman) as well as NCX itself] in addition to other factors will ultimately influence the current generated by the exchanger.

One of the major influences on NCX activity is the intracellular Ca^2+^ concentration. It is well known that cardiomyocytes isolated from failing hearts (with reduced ejection fraction) show altered Na^+^ and Ca^2+^ haemostasis. The modified Ca^2+^ handling is characterized by decreased Ca^2+^ transients, enhanced diastolic SR Ca^2+^ release and diminished SR Ca^2+^ reuptake, which all contribute to altered Ca^2+^ concentrations ‘seen’ by the NCX (Hasenfuss and Pieske, 2002; Kho et al., 2012; Luo and Anderson, 2013). Additionally, modeling studies have shown that that both dyadic and SR Ca^2+^ influence the appearance of DADs in addition to alterations in Ca^2+^ diffusion across the cell and Ca^2+^ uptake into the SR (Fink et al., 2011).

One of the first papers investigating Ca^2+^ sparks in myocytes from patients with HF indicated that alterations in the Ca^2+^ release mechanisms must be one of the mechanisms underlying EC coupling, in addition to alterations in SR Ca^2+^ load (Lindner et al., 2002). One of the driving forces behind this is the altered open probability of RyRs, which is governed by multiple factors, and has not been without controversy over the years (Dobrev and Wehrens, 2014). Interestingly, recent work has shown that stabilizing the RyR, using dantrolene, is able to prevent DADs in myocytes isolated from HF patients (Hartmann et al., 2017). Over the next few paragraphs, we will discuss how the major controllers of RyR stability, that also govern stretch and/or β-adrenergic signaling (namely CaMKII and ROS), can be affected in HF.

CaMKII phosphorylation of RyR appears to play an important role in arrhythmogenesis and sudden cardiac death in mice with HF (van Oort et al., 2010). Analysis from ventricular tissue from patients with either dilated or ischemic cardiomyopathy have shown that there is an increase in the levels of CaMKIIδ, the major isoform of CaMKII in the heart (Sossalla et al., 2010). Interestingly, single myocytes isolated from mice overexpressing CaMKIIδ are more liable to show DADs and spontaneous APs under β-adrenergic stimulation when compared to wild type mice (Sag et al., 2009). This increase in CaMKII seen in HF could directly promote arrhythmia formation by not only increasing diastolic Ca^2+^ leak via RyR phosphorylation, but also by promoting increases in the late Na^+^ current (Wagner et al., 2006), a current that has already been shown to be increased in HF, and incriminated in increased BVR and arrhythmia formation under these conditions (Maltsev et al., 2007).

The activity of CaMKII itself is under control of many different regulators, including ROS (as stated above and shown in **Figure 2**), which is detrimentally altered in HF. ROS also as having their own independent effects on RyR and other components of the Ca^2+^ handling machinery (dependent on the source of the ROS). In HF, just as in normal physiology, ROS has a number of different sources including NOX2, mitochondria and uncoupled NOS (Sag et al., 2013).

Interestingly, NOX2 expression and/or activity has also been shown to be increased in end-stage human HF in a number of studies, supporting the potential involvement of this pathway in the formation of ROS that may interfere with Ca^2+^ handling and lead to subsequent arrhythmias (Zhang M. et al., 2013). Furthermore, the elevated intracellular Na^+^ concentration seen in HF promotes the production of mitochondrial ROS (Kohlhaas et al., 2010; Viatchenko-Karpinski et al., 2014), which could ultimately lead to the potential for a vicious circle of proarrhythmic signaling via CaMKII.

Diseased hearts have been shown to have a significant increase in nNOS mRNA and protein expression (Damy et al., 2004). While on the other hand, several studies have provided evidence that NO production by eNOS is markedly diminished in HF, and an overexpression of eNOS has been shown to relieve cardiac dysfunction in a mouse model of HF (Katz et al., 1999; Jones et al., 2003; Damy et al., 2004). Under normal physiological conditions eNOS appears to decrease β-adrenergic responsiveness via inhibition of LTCC (Wang et al., 2008), therefore a reduction in this mechanism may be an additional driving force for β-adrenergic driven arrhythmias under these conditions. The overall increase in nNOS activity in HF, and the potentially altered signaling activity and targets (for example caveolae-associated molecules versus the RyR) resulting from the translocation of this molecule to the sarcolemma, may be important for deleterious Ca^2+^ handling and arrhythmia formation (Damy et al., 2004). Additionally, in HF it appears that NO production inducible NOS (iNOS) becomes of increased importance, although the role of this is currently less defined (Massion et al., 2003; Carnicer et al., 2013).

The activity of cAMP/PKA is tightly regulation by the activity of specific phosphodiesterases (PDEs) and protein phosphatases, however, the distribution of these is out of the scope of the present manuscript (see Guellich et al., 2014 for a review on this matter).

Finally, it is important to consider that the relative contribution of the different subtypes of β-ARs may also contribute to the increased arrhythmogenic phenotype observed in HF. As noted previously distinct pathways are associated with the different subtypes. Previous work has shown that the β_1_ subtype of adrenoreceptors are especially downregulated in HF, while the coupling of the receptors to Gs, presumably via increased activity of the receptor kinases GRK2 and/or GRK5, is altered (Lohse et al., 2003). Interestingly, β_2_-stimulation appears to be more arrhythmogenic in the failing heart when compared to the non-failing. Arrhythmogenesis appears to be driven by enhanced spontaneous SR Ca^2+^ release and aftercontractions, and is likely attributable, at least in part, to enhanced SR Ca^2+^ load secondary to PLB phosphorylation (DeSantiago et al., 2008). Away from the single cardiac myocyte, the requirement for β-Adrenergic stimulation to induce ectopic activity has also been shown in a human wedge preparation. Hearts from patients experiencing HF, exhibited ectopic beats and triggered activity in response to β_2_-stimulation. The authors of this study ascribe the increase in arrhythmogenic activity due to the enhancement of transmural differences between Ca^2+^ and APD, facilitating the formation of DADs (Lang et al., 2015).

All of the data that has been discussed up till now has not considered the potential for regional differences in remodeling, which may be triggered by various stimuli, stresses and strains sensed at different anatomical locations. Taking this into account, a recent paper interestingly showed that in a porcine model of myocardial infarction and HF, regional heterogeneities in arrhythmogenic remodeling do indeed exist. In this study, it was shown that changes in multiple currents lead to a shortening of AP at the border zone of the infarct, while APs recorded from the remote zone were prolonged. This will lead to a greater dispersion of repolarization across the ventricle, which could ultimately increase the arrhythmogenic substrate. Furthermore, these authors showed that cells isolated from the remote region showed DADs with a much higher frequency than in control, and amongst those cells, nearly half also showed triggered APs. Interestingly all HF-border cells showed DADs with over half showing triggered activity often with a superimposed EAD. In addition, inhibition of CaMKII decreased the occurrence of these DADs back to control levels, further indicating the importance of this multimodal signaling molecule in arrhythmia generation in this setting (Hegyi et al., 2018b). Although in this study these DADs were not induced by β-Adrenergic stimulation, but by burst pacing, one could also postulate that regional differences will also exist in terms of β-Adrenergic responsiveness in HF. In fact, a recent abstract from the Sipido group showed that in a pig model of MI, myocytes isolated from the peri-infarct region had a higher occurrence of isoproterenol induced DADs when compared to myocytes isolated from the region remote from the infarct (Dries et al., 2018b). These data pave the way for further research in this area.

### Local (Microdomain) Remodeling

So far, we have only focussed on global remodeling, however, in addition to heterogeneity seen across the ventricular wall as just described, the myocyte in itself is not homogeneous, especially when the micro-architecture of the myocyte is altered as is seen in HF. For these reasons, we must also consider local subcellular alterations. Given the improvements in imaging techniques, and experimental advances over recent years we have gained greater insights into how alterations in these ‘microdomains’ may influence arrhythmogenic outcomes in HF. Over the next paragraphs, we will discuss a number of studies that have been carried out in an attempt to elucidate how these microdomains may influence stretch- or β-adrenergic-induced arrhythmia in HF, with a view on targeted therapeutics (see the Section “Therapeutic Interventions”).

As noted previously, Ca^2+^ entry via the LTCC is the initial trigger for Ca^2+^ release from the SR, therefore it is important to discuss potential changes in this current in HF. At a global level, the majority of studies have shown that there is no alteration in whole cell Ca^2+^ current recorded from myocytes from HF patients or in animal models, although single channel studies have shown that the availability and open probability of the LTCC is increased in human HF myocytes (Beuckelmann and Erdmann, 1992; Mukherjee and Spinale, 1998; Schröder et al., 1998). However, over recent years a number of interesting observations have come to light indicating the location of LTCCs are different in HF myocytes. Alteration in the location of the LTCC will also have detrimental effects on the levels of Ca^2+^ the individual channels are exposed to. Therefore, Ca^2+^-dependent inactivation of the current as well as the dynamic modulation of the window current are likely to be altered in HF. Both of these changes will contribute to the formation of afterdepolarizations and can be influenced by β-adrenergic stimulation.

Using a rat model, combined with osmotic detubulation, Bryant et al. (2015) showed that although no differences in total I_CaL_ density was seen between ventricular myocytes isolated from animals that had undergone a coronary artery ligation, this lack of change resulted from differential effects at the cell surface and the T-tubules. I_CaL_ current density was decreased at the T-tubules while it was increased at the cell surface (Bryant et al., 2015).

An additional study from the group of Gorelik, using the super-resolution scanning patch-clamp technique showed similar findings. They elegantly showed that in both human and rat HF there was a redistribution of functional LTCCs from their physiological T-tubular location to the non-native crest of the sarcolemma. They went on to show that the open probability of these redistributed channels was dramatically increased, and the high open probability was linked to enhanced CaMKII modulation in the ‘new’ location. The current at these non-native channels resulted in an elevated I_CaL_ window current, which contributed to the development of EADs. This remained true when these data were fed-into a 3-dimensional left ventricle model illustrating that the phenomenon occurring at the single cell level has far reaching arrhythmogenic implications (Sanchez-Alonso et al., 2016). Interestingly, work carried out over 20 years ago indicated that there was a frequency dependent decrease in I_CaL_ in human dilated cardiomyopathy (Sipido et al., 1998). If this is to do with the altered LTCC microdomains, potentially due to CaMKII, remains to be seen, but should be the subject of future studies.

Localization of the LTCC to the T-tubules has previously been shown to be under control of the membrane scaffolding protein BIN1, with the knockdown of this protein leading to a reduction in surface LTCC and alterations in Ca^2+^ handling within the myocyte (Hong et al., 2010). Interestingly, BIN1 has been shown to be decreased in human HF as well as in a number of animal models, which may contribute to the alterations in patterns seen in LTCC localization (Hong et al., 2012; Caldwell et al., 2014). A more recent study has implicated that the β-adrenergic stimulation of BIN1 leads to reorganization of LTCC/RyR microdomains by also recruiting phosphorylated RyRs into the dyads. When BIN1 is downregulated, therefore, these phosphorylated RyRs may not be recruited in the dyad and arrhythmias may be promoted due to the defective Ca^2+^ handling (Fu et al., 2016).

Over 10 years ago, it was shown that in the failing heart there are a number of RyRs that become ‘orphaned’ from their LTCC counterpart in the T-tubule in spontaneously hypertensive rats (Song L.S. et al., 2006). These investigators used this model to show that this loss of coupling between the LTCC and RyR led to Ca^2+^ instability in the heart. More recently, Dries et al. (2018a) showed that in human myocytes isolated from HF patients, there were more non-coupled (to LTCC) RyRs which had more spontaneous activity than in non-HF. Hyperactivity of these non-coupled RyRs was reduced by CaMKII inhibition (Dries et al., 2018a). Previous work from the same group had illustrated that under healthy conditions, coupled (to LTCC) RyRs are distinctly modulated by CaMKII and ROS, while CaMKII and NOS1-dependent modulation of RyRs during β-adrenergic stimulation was also restricted to the dyadic cleft (Dries et al., 2013, 2016). The authors went on to use a pig model to further investigate alterations in coupled and non-coupled RyRs and their regulation in normal and pathophysiological conditions. However, after an MI, it was shown that under adrenergic stimulation using isoproterenol, Ca^2+^ waves were frequent and originated at non-coupled sites, generating larger NCX currents than in sham operated animals. Inhibition of CaMKII or mitochondrial-ROS scavenging reduced spontaneous Ca^2+^ waves, and improved excitation–contraction coupling, indicating that these could be interesting therapeutic targets (Dries et al., 2018a). A very recent paper corroborated the arrhythmogenic role of mitochondrial ROS in the formation of arrhythmias in a guinea pig model of non-ischemic HF (Dey et al., 2018). In this model, continuous telemetry recordings indicated a high frequency of premature ventricular complexes and spontaneous ventricular tachycardia/ventricular fibrillation in animals after aortic constriction and isoproterenol stimulation. Scavenging mitochondrial ROS using MitoTEMPO markedly suppressed arrhythmias as well as blunting QT prolongation and reducing QT variability. Taken all together these data indicate specific targeting of one source of ROS is adequate to reduce proarrhythmic outcomes.

In addition to alterations seen in the LTCC/RyR microdomain, and the control thereof, that may influence arrhythmogenesis we also need to consider alterations in the distribution of the different β-ARs themselves. One of the first reports investigating potential alterations was by Nikolaev et al. (2010). In that seminal study it was shown that, as opposed to the situation in healthy myocytes where β_1_-adrenergic receptors are widely distributed at the cell crest and β_2_-receptors (and their associated signaling pathways) are localized to the T-tubules, in HF β_2_-receptors were redistributed from the transverse tubules to the cell crest, leading to a change in β_2_-receptors associated compartmentation of cAMP (Nikolaev et al., 2010). These alterations lead to the β_2_-receptors acting more like β_1_-receptors and have detrimental effects on the cross-talk of adrenergic signaling and Ca^2+^ handling within the failing cell. Follow up studies have shown that the compartmentalization of the cAMP signaling from β_2_-receptors is governed by caveolin 3, a protein that regulates the number of caveolae in the myocyte. Alteration in the T-tubule structure, levels of caveolin 3 and junctophilin 2 appear to be time-dependent, and gradually alter the β_2_-signaling pathways. Furthermore, caveolin 3 overexpression in failing cells was able to restore, at least in part, the T-tubular location of the β_2_-receptors (Wright et al., 2014; Schobesberger et al., 2017).

Interestingly, an additional study utilizing a rabbit model, also showed that reintroduction of caveolin-3 was able to normalizes β-adrenergic-induced contractile responses in HF myocytes, while also showing that in HF β2-induced signaling gains access to myofilament which may contribute to abnormal PKA phosphorylation of troponin I and contractile dysfunction (Barbagallo et al., 2016). In addition, work from the Sacconi group has shown that while cells from HF myocytes respond to β-adrenergic stimulation, this is not the case at the T-tubules that do not conduct APs, where the alterations seen in response may be caused by a lack of electrical activity. These data provide an alternative, or an additional, mechanism for the alterations seen in HF (Crocini et al., 2016).

## Therapeutic Interventions

**FIGURE 3:**
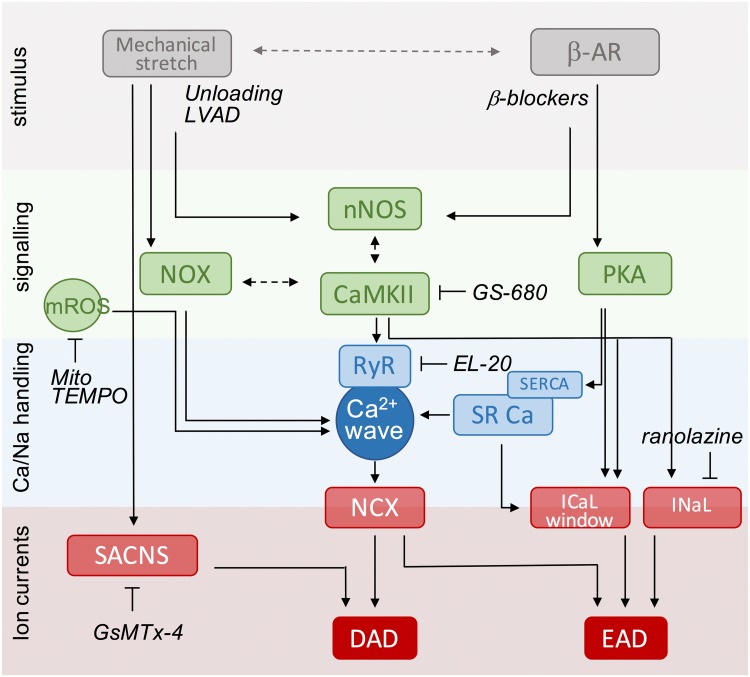
Scheme of key events in β-adrenergic and stretch signaling for the induction of afterdepolarizations as potential targets for anti-arrhythmic therapy. The central event in the generation of afterdepolarizations is a Ca^2+^ wave that can produce DAD or EAD depending on the timing of NCX activation during the cardiac cycle (diastole or systole). Mechanical stretch and β-adrenergic agonists activate protein kinases (PKA, CaMKII) and free radicals (ROS, NO) that increase RyR activity and/or SR Ca^2+^ load to produce Ca^2+^ sparks and waves. Likewise, they increase the activity of late Na^+^ and Ca^2+^ currents promoting EADs, directly via phosphorylation and redox modification, or indirectly via modulation by increased SR Ca^2+^ release secondary to enhanced SR Ca^2+^ load. Stretch-activated ion channels further destabilize membrane potential during stretch. Anti-arrhythmic strategies include targeting of upstream stressors, downstream signaling components, and Ca handling proteins or currents. Specific examples under current investigation are indicated and further discussed in the text. DAD, delayed afterdepolarizations; EAD, early afterdepolarizations; LVAD, left ventricular assistant device; NCX, Na^+^/Ca^2+^ exchanger; NO, nitric oxide; NOX, NADPH oxidase type; nNOS, neuronal nitric oxide synthase; PKA, protein kinase A; ROS, reactive oxygen species; RyR, ryanodine receptor; SACNS, stretch-activated non-selective cation currents; SR, sarcoplasmic reticulum; SERCA, SR Ca^2+^-ATPase.

Despite advances in our knowledge of the pathophysiology underlying HF over the last 10 years, as outlined above, only a limited number of compounds have become available for the treatment of this debilitating disease (Gordin and Fonarow, 2016). Given the information in previous sections, we could consider targeting primary stressors (i.e., β-adrenergic stimulation or mechanical load) or downstream pathways. In **Figure 3**, we have summarized the most important mechanisms of afterdepolarizations, highlighting relationships between stimuli, key signaling molecules and targeted Ca^2+^ handling proteins and ion currents. Based on this scheme, we will discuss a number of (potential) interventions over the next few paragraphs that could be utilized to reduce the arrhythmogenic burden in HF patients.

### Targeting Mechanical Load and Structural Remodeling

β-Blockers have been a mainstay of pharmacological treatment for HF for a number of years and are included in guidelines for treatment of HF, in part due to their antiarrhythmic properties (Funck-Brentano, 2006; Ponikowski et al., 2016). In addition to a reduction in β-adrenergic signaling that will be directly caused by β-blockers, they will also lead to a reduction of mechanical stressors that may also be responsible for the efficacy of β-blockers in preventing sudden cardiac death.

Not all β-blockers are equal, however, with some being selective at adrenoreceptor subtypes while others have additional actions at other ion channels. For instance, the antiarrhythmic effects of propranolol have also been ascribed to the potential of this compound to block Na^+^ channels (Fabritz et al., 2014). In long QT syndrome, for instance, it has been shown that propranolol has a significantly better QTc shortening effect compared to metoprolol and nadolol, and this led to a higher risk of cardiac events in symptomatic patients receiving metoprolol (Chockalingam et al., 2012). Therefore, given the information we currently have regarding the alteration in the number and location of subtypes of the β- receptors in HF, specific targeting of β-ARs, perhaps differing over the course of HF progression, may offer an interesting solution.

An additional way that mechanical load can be reduced is by the used of LVADs. Previous work has shown that the use of a LVADs together with the use of a specific pharmacological regimen, including the use of β-blockers, can lead to the sustained reversal of severe HF secondary to non-ischemic cardiomyopathy, even when the LVAD is explanted (Birks et al., 2006). Interestingly, it appeared that the alterations seen in the clinical phenotype in such patients was driven by modifications in ECC, and SR Ca^2+^ homeostasis in particular (Terracciano et al., 2004). More recent work has shown that mechanical unloading in a rat model (via heterotopic abdominal heart transplantation) reverses T-tubule remodeling, and normalizes local Ca^2+^ handling (Ibrahim et al., 2012).

Cardiac resynchronization therapy (CRT) is an alternative approach to alter mechanical loading and stretch and will assist to resynchronize ventricular wall motion. A recent meta-analysis indicated that this intervention may significantly reduce the risk of ventricular arrhythmias when compared with patients receiving ICDs. Interestingly the same study also showed that patients who were not-responsive to CRT may have had a significantly higher risk of ventricular arrhythmias (Saini et al., 2016). Furthermore, it was also shown that CRT was able to efficiently reduce subcellular heterogeneity of structure and function of RyRs and T-tubules in a canine model of HF, potentially due to the restoration of ventricular synchrony (Li et al., 2015).

Taking all of these data together it appears that alterations in mechanical load should be considered as a potential therapeutic strategy.

Another therapeutic strategy would be to potentially restrict remodeling in the first place. As previously mentioned, it appears there are a number of scaffolding proteins that are involved in the structural (and functional) remodeling of the Ca^2+^ microdomains in HF which could be the target for pharmaceutical intervention. For example, specific cardiac targeting of BIN1 could be an attractive option, although there are only limited data currently available and small molecules altering the activity of this protein are currently lacking. The potential for altering the T-tubule structure as a therapeutic intervention has recently been reviewed by Manfra et al. (2017).

### Direct Targeting

It could also be possible to directly target proteins involved in arrhythmia formation under conditions of enhanced stretch or intense ß-adrenergic stimulation. For example, the potential to target stretch activated channels was recently reviewed by White (2006), however, it is perhaps of note to mention GsMTx-4, which is a peptide isolated from tarantula venom. Mixed results have been obtained with GsMTx-4 to date, with one study showing the potential for reducing the number of premature ventricular beats in an ischemia/reperfusion mouse model, while an additional study indicated no benefit in a swine model (Barrabés et al., 2015; Wang et al., 2016). Further studies are required using this compound, and derivatives of it, to truly delineate the utility of inhibition of these channels in prevention of arrhythmogenesis in HF.

Furthermore, the blockade of the components of I_TI_ which actually contributes to afterdepolarization could also be an interesting target. Inhibition of NCX as a target has been discussed previously (Antoons et al., 2012), however, progress in this area has been hampered the lack by selective, and/or mode selective inhibitors. More recently a number of more selective agents have been developed, including ORM-10103 which have shown their utility in preventing Ca^2+^ overload induced arrhythmias (Nagy et al., 2014). However, in a rabbit HF model NCX inhibition with ORM-10103 reduced premature ventricular beats but was unable to suppress secondary Ca^2+^ rise or the occurrence of EADs (Chang et al., 2018).

Direct inhibition of ‘leaky’ RyRs could also be an interesting target to diminish proarrhythmic Ca^2+^ leak. A recent paper showed the utility of a tetracaine-derived compound, EL20, which was able to limit arrhythmogenic Ca^2+^ waves in a CPVT model by only limiting Ca^2+^ release from RyRs associated with calmodulin (Klipp et al., 2018). Should this have utility in HF should be the subject of further investigation.

An alternative approach would be to alter the activity of SERCA in HF. A number of pre-clinical studies showed that there was anti-arrhythmic potential for increasing SERCA activity in HF using adeno-associated virus technology (Kawase et al., 2008; Lyon et al., 2011). Despite initial positive outcomes in small trials (Jessup et al., 2011), larger clinical trials have been unable to show the benefit of this approach (Greenberg et al., 2016; Hulot et al., 2017). Despite these disappointing results, further studies are still required to investigate the true utility of this approach. Activation of SERCA using small molecules may also be potentially interesting approach, and recently istaroxime has become a compound of interest (Ferrandi et al., 2013). A clinical trial using this compound is currently ongoing (ClinicalTrials.gov Identifier: NCT02617446) with the results being highly anticipated.

### Targeting Downstream Modulation

An alternative avenue would be to target downstream signaling pathways that are involved. Perhaps one of the most investigated of these is the CaMKII pathway, perhaps due to the multimodal action of this kinase, and the fact that targeting this molecule may lead to selective modulation of proarrhythmic RyRs (Dries et al., 2018a). Interestingly it was recently shown that CaMKII activity persists even during chronic β-adrenergic blockade in HF, indicating that these two pathways could be targeted independently (Dewenter et al., 2017). To date, however, there are no clinically available CaMKII inhibitors, likely due to the lack of selective, bioavailable compounds. There are numerous reviews dealing with the potential of CaMKII inhibition as a potential treatment for HF, and for those reasons we will only discuss a couple of newer studies that have added fuel to the fire regarding the use of CaMKII inhibitors as antiarrhythmics (Swaminathan et al., 2012; Fischer et al., 2013; Westenbrink et al., 2013).

A recent study utilized the novel agent, GS-680, which is an ATP-dependent small molecule inhibitor of CaMKII, which appears to have good selectivity for CaMKII. In this study, this compound did not impair contractile function in failing human ventricular trabeculae and blunted the negative force-frequency relationship. Meanwhile, it increased the Ca^2+^ transient amplitude in isolated failing ventricular myocytes and reduced premature atrial contractions and afterdepolarizations in atrial cardiomyocytes (Lebek et al., 2018). Additionally, Dries et al. (2018a) recently showed that the specific CaMKII peptide inhibitor (AIP) significantly reduced the hyperactivity of RyRs in non-coupled regions without affecting spark frequency at coupled sites (Dries et al., 2018a). Finally, it has also been shown that inhibition of CaMKII is able to reduce the proarrhythmic effects of PDE-inhibitors, which act by increasing pools of cAMP (Bobin et al., 2016). If this is also true in HF should be the subject of future studies. If CaMKII inhibition will eventually function as an effective antiarrhythmic in HF still remains to be seen. While discussing CaMKII, it is also important to discuss the late Na^+^ current. Recent work has shown that inhibition of this current is able to suppress Ca^2+^ related arrhythmias by reducing CaMKII phosphorylation (Wei et al., 2017). Furthermore, ranolazine, an agent that inhibits both late Na^+^ and K^+^ channels has been shown to have antiarrhythmic effects in the intact heart in CHF and, interestingly, was not associated with drug-induced proarrhythmia (Frommeyer et al., 2012). Once again, further work is required to determine the true utility of late Na^+^ current inhibition under these circumstances.

Reactive oxygen species is one of the determinants of CaMKII activity, as well as having direct effects on Ca^2+^ handling proteins directly, so perhaps it is also possible to target ROS sources as an antiarrhythmic intervention. Interestingly the positive effect of the antioxidant vitamin C on the β-adrenergic response to dobutamine was blunted in HF patients, perhaps due to the global antioxidant properties of this agent (Mak and Newton, 2004), leading us to believe that a targeted approach may be required. The inhibition of NOX2 may be a promising approach although it may be necessary to avoid concurrent inhibition of other NADPHs which are required in other organ systems (Sag et al., 2014). Furthermore, supplementing with BH4, to ‘re-couple’ uncoupled NOS has been proposed (Moens et al., 2008), however, there have been issues with the bio-availability of this compound (see Bendall et al., 2014). The final source of ROS that could be an interesting target in mitochondrial ROS, and as noted earlier a number of studies have shown the utility of mitoTEMPO in reducing arrhythmia in HF (Dey et al., 2018; Dries et al., 2018a). The development of bioavailable safe tools to directly alter mitochondrial ROS production is still in its infancy and efforts should be concentrated to develop these.

Finally, targeting Epac has also been proposed as a potential target for arrhythmias driven by β-adrenergic stimulation, although there is discussion about its role. A recent study showed that treatment with an Epac activator, 8-CPT, enhanced the late Na^+^ current while inhibition of PKA (via PKI) did not affect the catecholamine induced increases in late Na^+^ current, indicating that Epac alone plays a crucial role in these arrhythmias (Dybkova et al., 2014; Fujita et al., 2017).

## Concluding Remarks

We still have a long way to go in treating ventricular arrhythmias that occur as a result of HF, however, concentrating on various microdomains within the cardiac myocytes that are under control of multiple pathways could be of great interest. Furthermore, linking the potential electro-mechanical and mechano-electro feedback loops could aid us in treating arrhythmias that occur as a result of this debilitating disease.

Is stretch sympathetic to HF? Only time and research will fully answer this question, but in the mean time we will have to be.

## Author Contributions

Both authors contributed to manuscript preparation and approved the final version of the manuscript.

## Conflict of Interest Statement

The authors declare that the research was conducted in the absence of any commercial or financial relationships that could be construed as a potential conflict of interest.

## Abbreviations

β-AR: β-adrenergic receptor
AC: adenyl cyclase
AP: action potential
BVR: beat-to-beat variability of repolarization
CaMKII: Ca^2+^/calmodulin dependent protein kinase II
cAMP: cyclic adenosine 3′,5′-monophosphate
Casq2: cardiac calsequestrin
CICR: calcium induced calcium release
DAD: delayed afterdepolarization
EAD: early afterdepolarization
ECC: excitation contraction coupling
Epac: exchange protein activated by cAMP
HF: heart failure
I_TI_: transient inward current
LTCC: L-type calcium channel
LVAD: left ventricular assist device
NCX: sodium/calcium exchanger
NO: nitric oxide
NOS: nitric oxide synthase
NOX2: NADPH oxidase 2
PI3K: phosphoinositide 3-kinase
PKA: protein kinase A
PLB: phospholamban
ROS: reactive oxygen species
RyR: ryanodine receptor
SACNS: stretch activated non-selective cation current
SERCA: SR Ca^2+^-ATPase
SR: sarcoplasmic reticulum
INaL: late sodium current

## References

[B1] Acsai K. Antoons G. Livshitz L. Rudy Y. Sipido K. R. (2011). Microdomain [Ca2^+^] near ryanodine receptors as reported by L-type Ca2^+^ and Na^+^/Ca2^+^ exchange currents. *J. Physiol.* 589 2569–2583. 10.1113/jphysiol.2010.202663 21486798 PMC3115826

[B2] Ahern G. P. Hsu S.-F. Klyachko V. A. Jackson M. B. (2000). Induction of persistent sodium current by exogenous and endogenous nitric oxide. *J. Biol. Chem.* 275 28810–28815. 10.1074/jbc.M003090200 10833522

[B3] Allen D. G. Kurihara S. (1982). The effects of muscle length on intracellular calcium transients in mammalian cardiac muscle. *J. Physiol.* 327 79–94. 10.1113/jphysiol.1982.sp014221 7120151 PMC1225098

[B4] Antoons G. Johnson D. M. Dries E. Santiago D. J. Ozdemir S. Lenaerts I. (2015). Calcium release near l-type calcium channels promotes beat-to-beat variability in ventricular myocytes from the chronic AV block dog. *J. Mol. Cell. Cardiol.* 89(Part B), 326–334. 10.1016/j.yjmcc.2015.10.008 26454162

[B5] Antoons G. Volders P. G. Stankovicova T. Bito V. Stengl M. Vos M. A. (2007). Window Ca2^+^ current and its modulation by Ca2^+^ release in hypertrophied cardiac myocytes from dogs with chronic atrioventricular block. *J. Physiol.* 579 147–160. 10.1113/jphysiol.2006.124222 17138604 PMC2075376

[B6] Antoons G. Willems R. Sipido K. R. (2012). Alternative strategies in arrhythmia therapy: evaluation of Na/Ca exchange as an anti-arrhythmic target. *Pharmacol. Ther.* 134 26–42. 10.1016/j.pharmthera.2011.12.001 22197992

[B7] Barbagallo F. Xu B. Reddy G. R. West T. Wang Q. Fu Q. (2016). Genetically encoded biosensors reveal PKA hyperphosphorylation on the myofilaments in rabbit heart failure. *Circ. Res.* 119 931–943. 10.1161/CIRCRESAHA.116.308964 27576469 PMC5307331

[B8] Barouch L. A. Harrison R. W. Skaf M. W. Rosas G. O. Cappola T. P. Kobeissi Z. A. (2002). Nitric oxide regulates the heart by spatial confinement of nitric oxide synthase isoforms. *Nature* 416 337–339. 10.1038/416005a 11907582

[B9] Barrabés J. A. Inserte J. Agulló L. Rodríguez-Sinovas A. Alburquerque-Béjar J. J. Garcia-Dorado D. (2015). Effects of the selective stretch-activated channel blocker GsMtx4 on stretch-induced changes in refractoriness in isolated rat hearts and on ventricular premature beats and arrhythmias after coronary occlusion in swine. *PLoS One* 10:e0125753. 10.1371/journal.pone.0125753 25938516 PMC4418727

[B10] Bendall J. K. Douglas G. McNeill E. Channon K. M. Crabtree M. J. (2014). Tetrahydrobiopterin in cardiovascular health and disease. *Antioxid. Redox Signal.* 20 3040–3077. 10.1089/ars.2013.5566 24294830 PMC4038990

[B11] Bers D. M. (2002). Cardiac excitation–contraction coupling. *Nature* 415 198–205. 10.1038/415198a 11805843

[B12] Bers D. M. Morotti S. (2014). Ca2^+^ current facilitation is CaMKII-dependent and has arrhythmogenic consequences. *Front. Pharmacol.* 5:144 10.3389/fphar.2014.00144 PMC4060732 24987371

[B13] Beuckelmann D. J. Erdmann E. (1992). Ca2^+^-currents and intracellular [Ca2^+^]i-transients in single ventricular myocytes isolated from terminally failing human myocardium. *Basic Res. Cardiol.* 87(Suppl. 1), 235–243. 1497571 10.1007/978-3-642-72474-9_19

[B14] Beuckelmann D. J. Näbauer M. Erdmann E. (1993). Alterations of K^+^ currents in isolated human ventricular myocytes from patients with terminal heart failure. *Circ. Res.* 73 379–385. 10.1161/01.RES.73.2.379 8330380

[B15] Biesmans L. Macquaide N. Heinzel F. R. Bito V. Smith G. L. Sipido K. R. (2011). Subcellular heterogeneity of ryanodine receptor properties in ventricular myocytes with low T-tubule density. *PLoS One* 6:e25100. 10.1371/journal.pone.0025100 22022376 PMC3192718

[B16] Birks E. J. Tansley P. D. Hardy J. George R. S. Bowles C. T. Burke M. (2006). Left ventricular assist device and drug therapy for the reversal of heart failure. *N. Engl. J. Med.* 355 1873–1884. 10.1056/NEJMoa053063 17079761

[B17] Bobin P. Varin A. Lefebvre F. Fischmeister R. Vandecasteele G. Leroy J. (2016). Calmodulin kinase II inhibition limits the pro-arrhythmic Ca2^+^ waves induced by cAMP-phosphodiesterase inhibitors. *Cardiovasc. Res.* 110 151–161. 10.1093/cvr/cvw027 26851245

[B18] Bristow M. R. Ginsburg R. Minobe W. Cubicciotti R. S. Sageman W. S. Lurie K. (1982). Decreased catecholamine sensitivity and beta-adrenergic-receptor density in failing human hearts. *N. Engl. J. Med.* 307 205–211. 10.1056/NEJM198207223070401 6283349

[B19] Bryant S. M. Kong C. H. Watson J. Cannell M. B. James A. F. Orchard C. H. (2015). Altered distribution of ICa impairs Ca release at the t-tubules of ventricular myocytes from failing hearts. *J. Mol. Cell. Cardiol.* 86 23–31. 10.1016/j.yjmcc.2015.06.012 26103619 PMC4564288

[B20] Burger D. E. Lu X. Lei M. Xiang F.-L. Hammoud L. Jiang M. (2009). Neuronal nitric oxide synthase protects against myocardial infarction-induced ventricular arrhythmia and mortality in mice. *Circulation* 120 1345–1354. 10.1161/CIRCULATIONAHA.108.846402 19770398

[B21] Burgoyne J. R. Mongue-Din H. Eaton P. Shah A. M. (2012). Redox signaling in cardiac physiology and pathology. *Circ. Res.* 111 1091–1106. 10.1161/CIRCRESAHA.111.255216 23023511

[B22] Calaghan S. C. Le Guennec J.-Y. White E. (2004). Cytoskeletal modulation of electrical and mechanical activity in cardiac myocytes. *Prog. Biophys. Mol. Biol.* 84 29–59. 10.1016/S0079-6107(03)00057-9 14642867

[B23] Caldwell J. L. Smith C. E. Taylor R. F. Kitmitto A. Eisner D. A. Dibb K. M. (2014). Dependence of cardiac transverse tubules on the BAR domain protein amphiphysin II (BIN-1). *Circ. Res.* 115 986–996. 10.1161/CIRCRESAHA.116.303448 25332206 PMC4274343

[B24] Cannell M. B. Kong C. H. (2012). Local control in cardiac E-C coupling. *J. Mol. Cell. Cardiol.* 52 298–303. 10.1016/j.yjmcc.2011.04.014 21586292

[B25] Carnicer R. Crabtree M. J. Sivakumaran V. Casadei B. Kass D. A. (2013). Nitric oxide synthases in heart failure. *Antioxid. Redox Signal.* 18 1078–1099. 10.1089/ars.2012.4824 22871241 PMC3567782

[B26] Carnicer R. Suffredini S. Liu X. Reilly S. Simon J. N. Surdo N. C. (2017). The subcellular localisation of neuronal nitric oxide synthase determines the downstream effects of no on myocardial function. *Cardiovasc. Res.* 10.1093/cvr/cvx002 [Epub ahead of print]. 28158509 PMC5408949

[B27] Catterall W. A. (2015). Regulation of cardiac calcium channels in the fight-or-flight response. *Curr. Mol. Pharmacol.* 8 12–21. 10.2174/1874467208666150507103417 25966697 PMC4664455

[B28] Cerrone M. Colombi B. Santoro M. di Barletta M. R. Scelsi M. Villani L. (2005). Bidirectional ventricular tachycardia and fibrillation elicited in a knock-in mouse model carrier of a mutation in the cardiac ryanodine receptor. *Circ. Res.* 96 e77–e82. 10.1161/01.RES.0000169067.51055.72 15890976

[B29] Cerrone M. Napolitano C. Priori S. G. (2009). Catecholaminergic polymorphic ventricular tachycardia: a paradigm to understand mechanisms of arrhythmias associated to impaired Ca2^+^ regulation. *Heart Rhythm* 6 1652–1659. 10.1016/j.hrthm.2009.06.033 19879546

[B30] Cerrone M. Noujaim S. F. Tolkacheva E. G. Talkachou A. O’Connell R. Berenfeld O. (2007). Arrhythmogenic mechanisms in a mouse model of catecholaminergic polymorphic ventricular tachycardia. *Circ. Res.* 101 1039–1048. 10.1161/CIRCRESAHA.107.148064 17872467 PMC2515360

[B31] Chang P.-C. Lu Y.-Y. Lee H.-L. Lin S.-F. Chu Y. Wen M.-S. (2018). Paradoxical effects of sodium-calcium exchanger inhibition on torsade de pointes and early afterdepolarization in a heart failure rabbit model. *J. Cardiovasc. Pharmacol.* 72 97–105. 10.1097/FJC.0000000000000598 29738372

[B32] Chen W.-W. Xiong X.-Q. Chen Q. Li Y.-H. Kang Y.-M. Zhu G.-Q. (2015). Cardiac sympathetic afferent reflex and its implications for sympathetic activation in chronic heart failure and hypertension. *Acta Physiol.* 213 778–794. 10.1111/apha.12447 25598170

[B33] Cheng H. Lederer W. J. (2008). Calcium sparks. *Physiol. Rev.* 88 1491–1545. 10.1152/physrev.00030.2007 18923188

[B34] Chen-Izu Y. Izu L. T. (2017). Mechano-chemo-transduction in cardiac myocytes. *J. Physiol.* 595 3949–3958. 10.1113/JP273101 28098356 PMC5471413

[B35] Chidsey C. A. Braunwald E. Morrow A. G. Mason D. T. (1963). Myocardial norepinephrine concentration in man. effects of reserpine and of congestive heart failure. *N. Engl. J. Med.* 269 653–658. 10.1056/NEJM196309262691302 14050968

[B36] Chockalingam P. Crotti L. Girardengo G. Johnson J. N. Harris K. M. van der Heijden J. F. (2012). Not all beta-blockers are equal in the management of long QT syndrome types 1 and 2: higher recurrence of events under metoprolol. *J. Am. Coll. Cardiol.* 60 2092–2099. 10.1016/j.jacc.2012.07.046 23083782 PMC3515779

[B37] Cingolani H. E. Pérez N. G. Cingolani O. H. Ennis I. L. (2013). The Anrep effect: 100 years later. *Am. J. Physiol. Heart Circ. Physiol.* 304 H175–H182. 10.1152/ajpheart.00508.2012 23161880

[B38] Cohn J. N. Ferrari R. Sharpe N. on Behalf of an International Forum on Cardiac Remodeling (2000). Cardiac remodeling—concepts and clinical implications: a consensus paper from an international forum on cardiac remodeling. *J. Am. Coll. Cardiol.* 35 569–582. 10.1016/S0735-1097(99)00630-0 10716457

[B39] Coste B. Mathur J. Schmidt M. Earley T. J. Ranade S. Petrus M. J. (2010). Piezo1 and Piezo2 are essential components of distinct mechanically activated cation channels. *Science* 330 55–60. 10.1126/science.1193270 20813920 PMC3062430

[B40] Craelius W. Chen V. el-Sherif N. (1988). Stretch activated ion channels in ventricular myocytes. *Biosci. Rep.* 8 407–414. 10.1007/BF01121637 2852974

[B41] Cranefield P. F. (1977). Action potentials, afterpotentials, and arrhythmias. *Circ. Res.* 41 415–423. 10.1161/01.RES.41.4.415 409566

[B42] Crocini C. Coppini R. Ferrantini C. Yan P. Loew L. M. Poggesi C. (2016). T-tubular electrical defects contribute to blunted β-adrenergic response in heart failure. *Int. J. Mol. Sci.* 17:E1471. 10.3390/ijms17091471 27598150 PMC5037749

[B43] Curran J. Hinton M. J. Ríos E. Bers D. M. Shannon T. R. (2007). β-adrenergic enhancement of sarcoplasmic reticulum calcium leak in cardiac myocytes is mediated by calcium/calmodulin-dependent protein kinase. *Circ. Res.* 100 391–398. 10.1161/01.RES.0000258172.74570.e6 17234966

[B44] Curran J. Tang L. Roof S. R. Velmurugan S. Millard A. Shonts S. (2014). Nitric oxide-dependent activation of CaMKII increases diastolic sarcoplasmic reticulum calcium release in cardiac myocytes in response to adrenergic stimulation. *PLoS One* 9:e87495. 10.1371/journal.pone.0087495 24498331 PMC3911966

[B45] Damy T. Ratajczak P. Shah A. M. Camors E. Marty I. Hasenfuss G. (2004). Increased neuronal nitric oxide synthase-derived NO production in the failing human heart. *Lancet* 363 1365–1367. 10.1016/S0140-6736(04)16048-0 15110495

[B46] Dangman K. H. Danilo P. Hordof A. J. Mary-Rabine L. Reder R. F. Rosen M. R. (1982). Electrophysiologic characteristics of human ventricular and Purkinje fibers. *Circulation* 65 362–368. 10.1161/01.CIR.65.2.362 7032748

[B47] DeSantiago J. Ai X. Islam M. Acuna G. Ziolo M. T. Bers D. M. (2008). Arrhythmogenic effects of β2-adrenergic stimulation in the failing heart are attributable to enhanced sarcoplasmic reticulum Ca load. *Circ. Res.* 102 1389–1397. 10.1161/CIRCRESAHA.107.169011 18467626 PMC2585979

[B48] Dewenter M. Neef S. Vettel C. Lämmle S. Beushausen C. Zelarayan L. C. (2017). Calcium/calmodulin-dependent protein kinase II activity persists during chronic β-adrenoceptor blockade in experimental and human heart failureclinical perspective. *Circ. Heart Fail.* 10:e003840. 10.1161/CIRCHEARTFAILURE.117.003840 28487342 PMC5479434

[B49] Dey S. DeMazumder D. Sidor A. Foster D. B. O’Rourke B. (2018). Mitochondrial ROS drive sudden cardiac death and chronic proteome remodeling in heart failure. *Circ. Res.* 123 356–371. 10.1161/CIRCRESAHA.118.312708 29898892 PMC6057154

[B50] Dobrev D. Wehrens X. H. (2014). Role of RyR2 phosphorylation in heart failure and arrhythmias: controversies around ryanodine receptor phosphorylation in cardiac disease. *Circ. Res.* 114 1311–1319. 10.1161/CIRCRESAHA.114.300568 24723656 PMC4008932

[B51] Doleschal B. Primessnig U. Wölkart G. Wolf S. Schernthaner M. Lichtenegger M. (2015). TRPC3 contributes to regulation of cardiac contractility and arrhythmogenesis by dynamic interaction with NCX1. *Cardiovasc. Res.* 106 163–173. 10.1093/cvr/cvv022 25631581 PMC4362401

[B52] Dries E. Bito V. Lenaerts I. Antoons G. Sipido K. R. Macquaide N. (2013). Selective modulation of coupled ryanodine receptors during microdomain activation of calcium/calmodulin-dependent kinase II in the dyadic cleft. *Circ. Res.* 113 1242–1252. 10.1161/CIRCRESAHA.113.301896 24081880

[B53] Dries E. Santiago D. J. Gilbert G. Lenaerts I. Vandenberk B. Nagaraju C. K. (2018a). Hyperactive ryanodine receptors in human heart failure and ischemic cardiomyopathy reside outside of couplons. *Cardiovasc. Res.* 114 1512–1524. 10.1093/cvr/cvy088 29668881 PMC6106102

[B54] Dries E. Vandenberk B. Gilbert G. Amoni M. Holemans P. Willems R. (2018b). P519Regional heterogeneity of hyperactive non-coupled ryanodine receptors makes the peri-infarct region more prone to triggered activities after myocardial infarction. *Cardiovasc. Res.* 114 S126–S127. 10.1093/cvr/cvy060.376

[B55] Dries E. Santiago D. J. Johnson D. M. Gilbert G. Holemans P. Korte S. M. (2016). Calcium/calmodulin-dependent kinase II and nitric oxide synthase 1-dependent modulation of ryanodine receptors during β-adrenergic stimulation is restricted to the dyadic cleft. *J. Physiol.* 594 5923–5939. 10.1113/JP271965 27121757 PMC5063942

[B56] Dybkova N. Wagner S. Backs J. Hund T. J. Mohler P. J. Sowa T. (2014). Tubulin polymerization disrupts cardiac β-adrenergic regulation of late INa. *Cardiovasc. Res.* 103 168–177. 10.1093/cvr/cvu120 24812278 PMC4133594

[B57] Eckberg D. L. Drabinsky M. Braunwald E. (1971). Defective cardiac parasympathetic control in patients with heart disease. *N. Engl. J. Med.* 285 877–883. 10.1056/NEJM197110142851602 4398792

[B58] Eder P. Molkentin J. D. (2011). TRPC channels as effectors of cardiac hypertrophy. *Circ. Res.* 108 265–272. 10.1161/CIRCRESAHA.110.225888 21252153

[B59] Erickson J. R. Joiner M. A. Guan X. Kutschke W. Yang J. Oddis C. V. (2008). A dynamic pathway for calcium-independent activation of CaMKII by methionine oxidation. *Cell* 133 462–474. 10.1016/j.cell.2008.02.048 18455987 PMC2435269

[B60] Fabritz L. Franz M. R. Carmeliet E. Kirchhof P. (2014). To the Editor–Propranolol, a β-adrenoreceptor blocker, prevents arrhythmias also by its sodium channel blocking effect. *Heart Rhythm* 11:e1. 10.1016/j.hrthm.2013.12.027 24361342

[B61] Faggioni M. Hwang H. S. van der Werf C. Nederend I. Kannankeril P. J. Wilde A. A. (2013). Accelerated sinus rhythm prevents catecholaminergic polymorphic ventricular tachycardia in mice and in patients. *Circ. Res.* 112 689–697. 10.1161/CIRCRESAHA.111.300076 23295832 PMC3601570

[B62] Farah C. Michel L. Y. M. Balligand J.-L. (2018). Nitric oxide signalling in cardiovascular health and disease. *Nat. Rev. Cardiol.* 15 292–316. 10.1038/nrcardio.2017.224 29388567

[B63] Fedida D. Noble D. Rankin A. C. Spindler A. J. (1987). The arrhythmogenic transient inward current iTI and related contraction in isolated guinea-pig ventricular myocytes. *J. Physiol.* 392 523–542. 10.1113/jphysiol.1987.sp016795 2451728 PMC1192319

[B64] Fernández-Velasco M. Rueda A. Rizzi N. Benitah J.-P. Colombi B. Napolitano C. (2009). Increased Ca2^+^ sensitivity of the ryanodine receptor mutant RyR2R4496C underlies catecholaminergic polymorphic ventricular tachycardia. *Circ. Res.* 104 201–209. 10.1161/CIRCRESAHA.108.177493 19096022 PMC2796688

[B65] Ferrandi M. Barassi P. Tadini-Buoninsegni F. Bartolommei G. Molinari I. Tripodi M. G. (2013). Istaroxime stimulates SERCA2a and accelerates calcium cycling in heart failure by relieving phospholamban inhibition. *Br. J. Pharmacol.* 169 1849–1861. 10.1111/bph.12278 23763364 PMC3753840

[B66] Fink M. Noble P. J. Noble D. (2011). Ca2^+^-induced delayed afterdepolarizations are triggered by dyadic subspace Ca2^+^ affirming that increasing SERCA reduces aftercontractions. *Am. J. Physiol. Heart Circ. Physiol.* 301 H921–H935. 10.1152/ajpheart.01055.2010 21666112 PMC3191104

[B67] Fischer T. H. Neef S. Maier L. S. (2013). The Ca-calmodulin dependent kinase II: a promising target for future antiarrhythmic therapies? *J. Mol. Cell. Cardiol.* 58 182–187. 10.1016/j.yjmcc.2012.11.003 23159442

[B68] Florea V. G. Mareyev V. Y. Samko A. N. Orlova I. A. Coats A. J. Belenkov Y. N. (1999). Left ventricular remodelling: common process in patients with different primary myocardial disorders. *Int. J. Cardiol.* 68 281–287. 10.1016/S0167-5273(98)00362-3 10213279

[B69] Forbes M. S. Sperelakis N. (1983). The membrane systems and cytoskeletal elements of mammalian myocardial cells. *Cell Muscle Motil.* 3 89–155. 10.1007/978-1-4615-9296-9_5 6231093

[B70] Fowler E. D. Kong C. H. T. Hancox J. C. Cannell M. B. (2018). Late Ca2^+^ sparks and ripples during the systolic Ca2^+^ transient in heart muscle cellsnovelty and significance. *Circ. Res.* 122 473–478. 10.1161/CIRCRESAHA.117.312257 29282211 PMC5796647

[B71] Franz M. R. Burkhoff D. Yue D. T. Sagawa K. (1989). Mechanically induced action potential changes and arrhythmia in isolated and in situ canine hearts. *Cardiovasc. Res.* 23 213–223. 10.1093/cvr/23.3.213 2590905

[B72] Frisk M. Ruud M. Espe E. K. S. Aronsen J. M. Røe Å. T. Zhang L. (2016). Elevated ventricular wall stress disrupts cardiomyocyte t-tubule structure and calcium homeostasis. *Cardiovasc. Res.* 112 443–451. 10.1093/cvr/cvw111 27226008 PMC5031949

[B73] Frommeyer G. Rajamani S. Grundmann F. Stypmann J. Osada N. Breithardt G. (2012). New insights into the beneficial electrophysiologic profile of ranolazine in heart failure: prevention of ventricular fibrillation with increased postrepolarization refractoriness and without drug-induced proarrhythmia. *J. Card. Fail.* 18 939–949. 10.1016/j.cardfail.2012.10.017 23207083

[B74] Fu Y. Shaw S. A. Naami R. Vuong C. L. Basheer W. A. Guo X. (2016). Isoproterenol promotes rapid ryanodine receptor movement to bridging integrator 1 (BIN1)-organized dyads. *Circulation* 133 388–397. 10.1161/CIRCULATIONAHA.115.018535 26733606 PMC4729615

[B75] Fujita T. Umemura M. Yokoyama U. Okumura S. Ishikawa Y. (2017). The role of Epac in the heart. *Cell. Mol. Life Sci.* 74 591–606. 10.1007/s00018-016-2336-5 27549789 PMC11107744

[B76] Funck-Brentano C. (2006). Beta-blockade in CHF: from contraindication to indication. *Eur. Heart J. Suppl.* 8 C19–C27. 10.1093/eurheartj/sul010

[B77] Garan A. R. Yuzefpolskaya M. Colombo P. C. Morrow J. P. Te-Frey R. Dano D. (2013). Ventricular arrhythmias and implantable cardioverter-defibrillator therapy in patients with continuous-flow left ventricular assist devices. *J. Am. Coll. Cardiol.* 61 2542–2550. 10.1016/j.jacc.2013.04.020 23643502

[B78] Gill J. S. McKenna W. J. Camm A. J. (1995). Free radicals irreversibly decrease Ca2^+^ currents in isolated guinea-pig ventricular myocytes. *Eur. J. Pharmacol.* 292 337–340. 7796875 10.1016/0926-6917(95)90042-x

[B79] Gómez A. M. Ruiz-Hurtado G. Benitah J.-P. Domínguez-Rodríguez A. (2013). Ca(2^+^) fluxes involvement in gene expression during cardiac hypertrophy. *Curr. Vasc. Pharmacol.* 11 497–506. 10.2174/1570161111311040013 23905644

[B80] González D. R. Fernández I. C. Ordenes P. P. Treuer A. V. Eller G. Boric M. P. (2008). Differential role of S-nitrosylation and the NO-cGMP-PKG pathway in cardiac contractility. *Nitric Oxide* 18 157–167. 10.1016/j.niox.2007.09.086 18023373

[B81] Gordin J. S. Fonarow G. C. (2016). New medications for heart failure. *Trends Cardiovasc. Med.* 26 485–492. 10.1016/j.tcm.2016.02.008 27038558 PMC4958495

[B82] Gottlieb P. Folgering J. Maroto R. Raso A. Wood T. G. Kurosky A. (2008). Revisiting TRPC1 and TRPC6 mechanosensitivity. *Pflugers Arch.* 455 1097–1103. 10.1007/s00424-007-0359-3 17957383

[B83] Gottlieb P. A. (2017). A tour de force: the discovery, properties, and function of piezo channels. *Curr. Top. Membr.* 79 1–36. 10.1016/bs.ctm.2016.11.007 28728814

[B84] Greenberg B. Butler J. Felker G. M. Ponikowski P. Voors A. A. Desai A. S. (2016). Calcium upregulation by percutaneous administration of gene therapy in patients with cardiac disease (CUPID 2): a randomised, multinational, double-blind, placebo-controlled, phase 2b trial. *Lancet* 387 1178–1186. 10.1016/S0140-6736(16)00082-9 26803443

[B85] Guellich A. Mehel H. Fischmeister R. (2014). Cyclic AMP synthesis and hydrolysis in the normal and failing heart. *Pflugers Arch.* 466 1163–1175. 10.1007/s00424-014-1515-1 24756197

[B86] Guo A. Zhang C. Wei S. Chen B. Song L.-S. (2013). Emerging mechanisms of T-tubule remodelling in heart failure. *Cardiovasc. Res.* 98 204–215. 10.1093/cvr/cvt020 23393229 PMC3697065

[B87] Guo J. Duff H. J. (2006). Calmodulin kinase II accelerates L-type Ca2^+^ current recovery from inactivation and compensates for the direct inhibitory effect of [Ca2^+^]i in rat ventricular myocytes. *J. Physiol.* 574 509–518. 10.1113/jphysiol.2006.109199 16627565 PMC1817774

[B88] Guo T. Zhang T. Ginsburg K. S. Mishra S. Brown J. H. Bers D. M. (2012). CaMKIIδC slows [Ca]i decline in cardiac myocytes by promoting Ca sparks. *Biophys. J.* 102 2461–2470. 10.1016/j.bpj.2012.04.015 22713561 PMC3368151

[B89] Gutierrez D. A. Fernandez-Tenorio M. Ogrodnik J. Niggli E. (2013). NO-dependent CaMKII activation during β-adrenergic stimulation of cardiac muscle. *Cardiovasc. Res.* 100 392–401. 10.1093/cvr/cvt201 23963842

[B90] Hansen D. E. Craig C. S. Hondeghem L. M. (1990). Stretch-induced arrhythmias in the isolated canine ventricle. Evidence for the importance of mechanoelectrical feedback. *Circulation* 81 1094–1105. 10.1161/01.CIR.81.3.1094 1689619

[B91] Hartmann N. Pabel S. Herting J. Schatter F. Renner A. Gummert J. (2017). Antiarrhythmic effects of dantrolene in human diseased cardiomyocytes. *Heart Rhythm* 14 412–419. 10.1016/j.hrthm.2016.09.014 27650424

[B92] Hasenfuss G. Pieske B. (2002). Calcium cycling in congestive heart failure. *J. Mol. Cell. Cardiol.* 34 951–969. 10.1006/jmcc.2002.2037 12234765

[B93] Hegyi B. Bossuyt J. Ginsburg K. S. Mendoza L. M. Talken L. Ferrier W. T. (2018a). Altered repolarization reserve in failing rabbit ventricular myocytes: calcium and β-adrenergic effects on delayed- and inward-rectifier potassium currents. *Circ. Arrhythm. Electrophysiol.* 11:e005852. 10.1161/CIRCEP.117.005852 29437761 PMC5813707

[B94] Hegyi B. Bossuyt J. Griffiths L. G. Shimkunas R. Coulibaly Z. Jian Z. (2018b). Complex electrophysiological remodeling in postinfarction ischemic heart failure. *Proc. Natl. Acad. Sci. U.S.A.* 115 E3036–E3044. 10.1073/pnas.1718211115 29531045 PMC5879679

[B95] Heinzel F. R. MacQuaide N. Biesmans L. Sipido K. (2011). Dyssynchrony of Ca2^+^ release from the sarcoplasmic reticulum as subcellular mechanism of cardiac contractile dysfunction. *J. Mol. Cell. Cardiol.* 50 390–400. 10.1016/j.yjmcc.2010.11.008 21075114

[B96] Hong T.-T. Cogswell R. James C. A. Kang G. Pullinger C. R. Malloy M. J. (2012). Plasma BIN1 correlates with heart failure and predicts arrhythmia in patients with arrhythmogenic right ventricular cardiomyopathy. *Heart Rhythm* 9 961–967. 10.1016/j.hrthm.2012.01.024 22300662 PMC3349006

[B97] Hong T.-T. Smyth J. W. Gao D. Chu K. Y. Vogan J. M. Fong T. S. (2010). BIN1 localizes the L-type calcium channel to cardiac T-tubules. *PLoS Biol.* 8:e1000312. 10.1371/journal.pbio.1000312 20169111 PMC2821894

[B98] Houser S. R. Molkentin J. D. (2008). Does contractile Ca2^+^ control calcineurin-NFAT signaling and pathological hypertrophy in cardiac myocytes? *Sci. Signal.* 1:e31 10.1126/scisignal.125pe31 PMC2680250 18577756

[B99] Hulot J.-S. Salem J.-E. Redheuil A. Collet J.-P. Varnous S. Jourdain P. (2017). Effect of intracoronary administration of AAV1/SERCA2a on ventricular remodelling in patients with advanced systolic heart failure: results from the AGENT-HF randomized phase 2 trial. *Eur. J. Heart Fail.* 19 1534–1541. 10.1002/ejhf.826 28393439

[B100] Ibrahim M. Navaratnarajah M. Siedlecka U. Rao C. Dias P. Moshkov A. V. (2012). Mechanical unloading reverses transverse tubule remodelling and normalizes local Ca2^+^-induced Ca2^+^ release in a rodent model of heart failure. *Eur. J. Heart Fail.* 14 571–580. 10.1093/eurjhf/hfs038 22467752 PMC3359860

[B101] Inoue R. Jian Z. Kawarabayashi Y. (2009). Mechanosensitive TRP channels in cardiovascular pathophysiology. *Pharmacol. Ther.* 123 371–385. 10.1016/j.pharmthera.2009.05.009 19501617

[B102] Iribe G. Ward C. W. Camelliti P. Bollensdorff C. Mason F. Burton R. A. (2009). Axial stretch of rat single ventricular cardiomyocytes causes an acute and transient increase in Ca2^+^ spark rate. *Circ. Res.* 104 787–795. 10.1161/CIRCRESAHA.108.193334 19197074 PMC3522525

[B103] Janse M. J. (2004). Electrophysiological changes in heart failure and their relationship to arrhythmogenesis. *Cardiovasc. Res.* 61 208–217. 10.1016/j.cardiores.2003.11.018 14736537

[B104] January C. T. Riddle J. M. (1989). Early afterdepolarizations: mechanism of induction and block. A role for L-type Ca2^+^ current. *Circ. Res.* 64 977–990. 10.1161/01.RES.64.5.977 2468430

[B105] Jessup M. Greenberg B. Mancini D. Cappola T. Pauly D. F. Jaski B. (2011). Calcium upregulation by percutaneous administration of gene therapy in cardiac disease (CUPID): a phase 2 trial of intracoronary gene therapy of sarcoplasmic reticulum Ca2^+^ -ATPase in patients with advanced heart failure. *Circulation* 124 304–313. 10.1161/CIRCULATIONAHA.111.022889 21709064 PMC5843948

[B106] Jian Z. Han H. Zhang T. Puglisi J. Izu L. T. Shaw J. A. (2014). Mechanochemotransduction during cardiomyocyte contraction is mediated by localized nitric oxide signaling. *Sci. Signal.* 7:ra27. 10.1126/scisignal.2005046 24643800 PMC4103414

[B107] Johnson D. M. Heijman J. Bode E. F. Greensmith D. J. van der Linde H. Abi-Gerges N. (2013). Diastolic spontaneous calcium release from the sarcoplasmic reticulum increases beat-to-beat variability of repolarization in canine ventricular myocytes after β-adrenergic stimulation. *Circ. Res.* 112 246–256. 10.1161/CIRCRESAHA.112.275735 23149594

[B108] Johnson D. M. Heijman J. Pollard C. E. Valentin J.-P. Crijns H. J. G. M. Abi-Gerges N. (2010). I(Ks) restricts excessive beat-to-beat variability of repolarization during beta-adrenergic receptor stimulation. *J. Mol. Cell. Cardiol.* 48 122–130. 10.1016/j.yjmcc.2009.08.033 19744496

[B109] Jones S. P. Greer J. J. M. van Haperen R. Duncker D. J. de Crom R. Lefer D. J. (2003). Endothelial nitric oxide synthase overexpression attenuates congestive heart failure in mice. *Proc. Natl. Acad. Sci. U.S.A.* 100 4891–4896. 10.1073/pnas.0837428100 12676984 PMC153651

[B110] Jost N. Virág L. Bitay M. Takács J. Lengyel C. Biliczki P. (2005). Restricting excessive cardiac action potential and QT prolongation: a vital role for IKs in human ventricular muscle. *Circulation* 112 1392–1399. 10.1161/CIRCULATIONAHA.105.550111 16129791

[B111] Kashimura T. Briston S. J. Trafford A. W. Napolitano C. Priori S. G. Eisner D. A. (2010). In the RyR2R4496C mouse model of CPVT, β-adrenergic stimulation induces Ca waves by increasing SR Ca content and not by decreasing the threshold for Ca waves novelty and significance. *Circ. Res.* 107 1483–1489. 10.1161/CIRCRESAHA.110.227744 20966392

[B112] Kassmann M. Hansel A. Leipold E. Birkenbeil J. Lu S.-Q. Hoshi T. (2008). Oxidation of multiple methionine residues impairs rapid sodium channel inactivation. *Pflugers Arch.* 456 1085–1095. 10.1007/s00424-008-0477-6 18369661 PMC2913308

[B113] Katz S. D. Khan T. Zeballos G. A. Mathew L. Potharlanka P. Knecht M. (1999). Decreased activity of the L-arginine-nitric oxide metabolic pathway in patients with congestive heart failure. *Circulation* 99 2113–2117. 10.1161/01.CIR.99.16.2113 10217650

[B114] Kawase Y. Ly H. Q. Prunier F. Lebeche D. Shi Y. Jin H. (2008). Reversal of cardiac dysfunction after long-term expression of SERCA2a by gene transfer in a pre-clinical model of heart failure. *J. Am. Coll. Cardiol.* 51 1112–1119. 10.1016/j.jacc.2007.12.014 18342232

[B115] Kehat I. Molkentin J. D. (2010). Molecular pathways underlying cardiac remodeling during pathophysiologic stimulation. *Circulation* 122 2727–2735. 10.1161/CIRCULATIONAHA.110.942268 21173361 PMC3076218

[B116] Khairallah R. J. Shi G. Sbrana F. Prosser B. L. Borroto C. Mazaitis M. J. (2012). Microtubules underlie dysfunction in duchenne muscular dystrophy. *Sci. Signal.* 5:ra56. 10.1126/scisignal.2002829 22871609 PMC3835660

[B117] Kho C. Lee A. Hajjar R. J. (2012). Altered sarcoplasmic reticulum calcium cycling–targets for heart failure therapy. *Nat. Rev. Cardiol.* 9 717–733. 10.1038/nrcardio.2012.145 23090087 PMC3651893

[B118] Klipp R. C. Li N. Wang Q. Word T. A. Sibrian-Vazquez M. Strongin R. M. (2018). EL20, a potent antiarrhythmic compound, selectively inhibits calmodulin-deficient ryanodine receptor type 2. *Heart Rhythm* 15 578–586. 10.1016/j.hrthm.2017.12.017 29248564 PMC5879004

[B119] Kohlhaas M. Liu T. Knopp A. Zeller T. Ong M. F. Böhm M. (2010). Elevated cytosolic Na^+^ increases mitochondrial formation of reactive oxygen species in failing cardiac myocytes. *Circulation* 121 1606–1613. 10.1161/CIRCULATIONAHA.109.914911 20351235 PMC2946079

[B120] Kokkonen K. Kass D. A. (2017). Nanodomain regulation of cardiac cyclic nucleotide signaling by phosphodiesterases. *Annu. Rev. Pharmacol. Toxicol.* 57 455–479. 10.1146/annurev-pharmtox-010716-104756 27732797

[B121] Koumi S. Backer C. L. Arentzen C. E. Sato R. (1995). beta-Adrenergic modulation of the inwardly rectifying potassium channel in isolated human ventricular myocytes. Alteration in channel response to beta-adrenergic stimulation in failing human hearts. *J. Clin. Invest.* 96 2870–2881. 10.1172/JCI118358 8675658 PMC185998

[B122] Kuwahara K. Wang Y. McAnally J. Richardson J. A. Bassel-Duby R. Hill J. A. (2006). TRPC6 fulfills a calcineurin signaling circuit during pathologic cardiac remodeling. *J. Clin. Invest.* 116 3114–3126. 10.1172/JCI27702 17099778 PMC1635163

[B123] Landstrom A. P. Dobrev D. Wehrens X. H. T. (2017). Calcium signaling and cardiac arrhythmias. *Circ. Res.* 120 1969–1993. 10.1161/CIRCRESAHA.117.310083 28596175 PMC5607780

[B124] Lang D. Holzem K. Kang C. Xiao M. Hwang H. J. Ewald G. A. (2015). Arrhythmogenic remodeling of β2 versus β1 adrenergic signaling in the human failing heart. *Circ. Arrhythm. Electrophysiol.* 8 409–419. 10.1161/CIRCEP.114.002065 25673629 PMC4608687

[B125] Lang D. Sato D. Jiang Y. Ginsburg K. S. Ripplinger C. M. Bers D. M. (2017). Calcium-dependent arrhythmogenic foci created by weakly coupled myocytes in the failing heart. *Circ. Res.* 121 1379–1391. 10.1161/CIRCRESAHA.117.312050 28970285 PMC5722688

[B126] Lazzara R. Marchi S. (1989). “Electrophysiologic mechanisms for the generation of arrhythmias with adrenergic stimulation,” in *Adrenergic System and Ventricular Arrhythmias in Myocardial Infarction*, eds Brachmann J. Schömig A. (Berlin: Springer), 231–238. 10.1007/978-3-642-74317-7_19

[B127] Lebek S. Plößl A. Baier M. Mustroph J. Tarnowski D. Lücht C. M. (2018). The novel CaMKII inhibitor GS-680 reduces diastolic SR Ca leak and prevents CaMKII-dependent pro-arrhythmic activity. *J. Mol. Cell. Cardiol.* 118 159–168. 10.1016/j.yjmcc.2018.03.020 29614261

[B128] Lederer W. J. Tsien R. W. (1976). Transient inward current underlying arrhythmogenic effects of cardiotonic steroids in Purkinje fibres. *J. Physiol.* 263 73–100. 10.1113/jphysiol.1976.sp011622 1018270 PMC1307691

[B129] Li G.-R. Lau C.-P. Ducharme A. Tardif J.-C. Nattel S. (2002). Transmural action potential and ionic current remodeling in ventricles of failing canine hearts. *Am. J. Physiol. Heart Circ. Physiol.* 283 H1031–H1041. 10.1152/ajpheart.00105.2002 12181133

[B130] Li H. Lichter J. G. Seidel T. Tomaselli G. F. Bridge J. H. B. Sachse F. B. (2015). Cardiac resynchronization therapy reduces subcellular heterogeneity of ryanodine receptors, T-tubules, and Ca2^+^ sparks produced by dyssynchronous heart failure. *Circ. Heart Fail.* 8 1105–1114. 10.1161/CIRCHEARTFAILURE.115.002352 26294422 PMC4651794

[B131] Liang J. Huang B. Yuan G. Chen Y. Liang F. Zeng H. (2017). Stretch-activated channel Piezo1 is up-regulated in failure heart and cardiomyocyte stimulated by AngII. *Am. J. Transl. Res.* 9 2945–2955. 28670382 PMC5489894

[B132] Lindner M. Brandt M. C. Sauer H. Hescheler J. Böhle T. Beuckelmann D. J. (2002). Calcium sparks in human ventricular cardiomyocytes from patients with terminal heart failure. *Cell Calcium* 31 175–182. 10.1054/ceca.2002.0272 12027382

[B133] Liu M. Liu H. Dudley S. C. (2010). Reactive oxygen species originating from mitochondria regulate the cardiac sodium channel. *Circ. Res.* 107 967–974. 10.1161/CIRCRESAHA.110.220673 20724705 PMC2955818

[B134] Liu N. Colombi B. Memmi M. Zissimopoulos S. Rizzi N. Negri S. (2006). Arrhythmogenesis in catecholaminergic polymorphic ventricular tachycardia: insights from a RyR2 R4496C knock-in mouse model. *Circ. Res.* 99 292–298. 10.1161/01.RES.0000235869.50747.e1 16825580

[B135] Lohse M. J. Engelhardt S. Eschenhagen T. (2003). What is the role of β-adrenergic signaling in heart failure? *Circ. Res.* 93 896–906. 10.1161/01.RES.0000102042.83024.CA 14615493

[B136] Luo M. Anderson M. E. (2013). Mechanisms of altered Ca2^+^ handling in heart failure. *Circ. Res.* 113 690–708. 10.1161/CIRCRESAHA.113.301651 23989713 PMC4080816

[B137] Lyon A. R. Bannister M. L. Collins T. Pearce E. Sepehripour A. H. Dubb S. S. (2011). SERCA2a gene transfer decreases sarcoplasmic reticulum calcium leak and reduces ventricular arrhythmias in a model of chronic heart failure. *Circ. Arrhythm. Electrophysiol.* 4 362–372. 10.1161/CIRCEP.110.961615 21406682 PMC3119354

[B138] Lyon A. R. MacLeod K. T. Zhang Y. Garcia E. Kanda G. K. Lab M. J. (2009). Loss of T-tubules and other changes to surface topography in ventricular myocytes from failing human and rat heart. *Proc. Natl. Acad. Sci. U.S.A.* 106 6854–6859. 10.1073/pnas.0809777106 19342485 PMC2672472

[B139] Maier L. S. Bers D. M. (2007). Role of Ca2^+^/calmodulin-dependent protein kinase (CaMK) in excitation-contraction coupling in the heart. *Cardiovasc. Res.* 73 631–640. 10.1016/j.cardiores.2006.11.005 17157285

[B140] Mak S. Newton G. E. (2004). Redox modulation of the inotropic response to dobutamine is impaired in patients with heart failure. *Am. J. Physiol. Heart Circ. Physiol.* 286 H789–H795. 10.1152/ajpheart.00633.2003 14551049

[B141] Makarewich C. A. Zhang H. Davis J. Correll R. N. Trappanese D. M. Hoffman N. E. (2014). Transient receptor potential channels contribute to pathological structural and functional remodeling after myocardial infarction. *Circ. Res.* 115 567–580. 10.1161/CIRCRESAHA.115.303831 25047165 PMC4149870

[B142] Malliani A. Recordati G. Schwartz P. J. (1973). Nervous activity of afferent cardiac sympathetic fibres with atrial and ventricular endings. *J. Physiol.* 229 457–469. 10.1113/jphysiol.1973.sp010147 4724832 PMC1350316

[B143] Maltsev V. A. Silverman N. Sabbah H. N. Undrovinas A. I. (2007). Chronic heart failure slows late sodium current in human and canine ventricular myocytes: implications for repolarization variability. *Eur. J. Heart Fail.* 9 219–227. 10.1016/j.ejheart.2006.08.007 17067855 PMC1847560

[B144] Manfra O. Frisk M. Louch W. E. (2017). Regulation of cardiomyocyte T-tubular structure: opportunities for therapy. *Curr. Heart Fail. Rep.* 14 167–178. 10.1007/s11897-017-0329-9 28447290 PMC5423965

[B145] Marban E. Robinson S. W. Wier W. G. (1986). Mechanisms of arrhythmogenic delayed and early afterdepolarizations in ferret ventricular muscle. *J. Clin. Invest.* 78 1185–1192. 10.1172/JCI112701 3771791 PMC423803

[B146] Marx S. O. Reiken S. Hisamatsu Y. Jayaraman T. Burkhoff D. Rosemblit N. (2000). PKA phosphorylation dissociates FKBP12.6 from the calcium release channel (ryanodine receptor): defective regulation in failing hearts. *Cell* 101 365–376. 10.1016/S0092-8674(00)80847-8 10830164

[B147] Massion P. B. Feron O. Dessy C. Balligand J.-L. (2003). Nitric oxide and cardiac function: ten years after, and continuing. *Circ. Res.* 93 388–398. 10.1161/01.RES.0000088351.58510.21 12958142

[B148] Miura M. Taguchi Y. Nagano T. Sasaki M. Handoh T. Shindoh C. (2015). Effect of myofilament Ca(2^+^) sensitivity on Ca(2^+^) wave propagation in rat ventricular muscle. *J. Mol. Cell. Cardiol.* 84 162–169. 10.1016/j.yjmcc.2015.04.027 25953256

[B149] Miura M. Wakayama Y. Endoh H. Nakano M. Sugai Y. Hirose M. (2008). Spatial non-uniformity of excitation-contraction coupling can enhance arrhythmogenic-delayed afterdepolarizations in rat cardiac muscle. *Cardiovasc. Res.* 80 55–61. 10.1093/cvr/cvn162 18558629

[B150] Moens A. L. Takimoto E. Tocchetti C. G. Chakir K. Bedja D. Cormaci G. (2008). Reversal of cardiac hypertrophy and fibrosis from pressure overload by tetrahydrobiopterin: efficacy of recoupling nitric oxide synthase as a therapeutic strategy. *Circulation* 117 2626–2636. 10.1161/CIRCULATIONAHA.107.737031 18474817 PMC2614930

[B151] Morita H. Suzuki G. Haddad W. Mika Y. Tanhehco E. J. Sharov V. G. (2003). Cardiac contractility modulation with nonexcitatory electric signals improves left ventricular function in dogs with chronic heart failure. *J. Card. Fail.* 9 69–75. 10.1054/jcaf.2003.8 12612875

[B152] Morita N. Sovari A. A. Xie Y. Fishbein M. C. Mandel W. J. Garfinkel A. (2009). Increased susceptibility of aged hearts to ventricular fibrillation during oxidative stress. *Am. J. Physiol. Heart Circ. Physiol.* 297 H1594–H1605. 10.1152/ajpheart.00579.2009 19767530 PMC2781379

[B153] Mukherjee R. Spinale F. G. (1998). L-type calcium channel abundance and function with cardiac hypertrophy and failure: a review. *J. Mol. Cell. Cardiol.* 30 1899–1916. 10.1006/jmcc.1998.0755 9799645

[B154] Muralidharan P. Szappanos H. C. Ingley E. Hool L. (2016). Evidence for redox sensing by a human cardiac calcium channel. *Sci. Rep.* 6:19067. 10.1038/srep19067 26750869 PMC4707475

[B155] Myles R. C. Wang L. Bers D. M. Ripplinger C. M. (2015). Decreased inward rectifying K^+^ current and increased ryanodine receptor sensitivity synergistically contribute to sustained focal arrhythmia in the intact rabbit heart. *J. Physiol.* 593 1479–1493. 10.1113/jphysiol.2014.279638 25772297 PMC4376425

[B156] Nagy N. Kormos A. Kohajda Z. Szebeni Á. Szepesi J. Pollesello P. (2014). Selective Na^+^/Ca2^+^ exchanger inhibition prevents Ca2^+^ overload-induced triggered arrhythmias. *Br. J. Pharmacol.* 171 5665–5681. 10.1111/bph.12867 25073832 PMC4290709

[B157] Nattel S. Maguy A. Le Bouter S. Yeh Y.-H. (2007). Arrhythmogenic ion-channel remodeling in the heart: heart failure, myocardial infarction, and atrial fibrillation. *Physiol. Rev.* 87 425–456. 10.1152/physrev.00014.2006 17429037

[B158] Neco P. Rose B. Huynh N. Zhang R. Bridge J. H. B. Philipson K. D. (2010). Sodium-calcium exchange is essential for effective triggering of calcium release in mouse heart. *Biophys. J.* 99 755–764. 10.1016/j.bpj.2010.04.071 20682252 PMC2913203

[B159] Nerbonne J. M. Kass R. S. (2005). Molecular physiology of cardiac repolarization. *Physiol. Rev.* 85 1205–1253. 10.1152/physrev.00002.2005 16183911

[B160] Neves J. S. Leite-Moreira A. M. Neiva-Sousa M. Almeida-Coelho J. Castro-Ferreira R. Leite-Moreira A. F. (2015). Acute myocardial response to stretch: what we (don’t) know. *Front. Physiol.* 6:408 10.3389/fphys.2015.00408 PMC4700209 26779036

[B161] Nikolaev V. O. Bünemann M. Schmitteckert E. Lohse M. J. Engelhardt S. (2006). Cyclic AMP imaging in adult cardiac myocytes reveals far-reaching β1-adrenergic but locally confined β2-adrenergic receptor–mediated signaling. *Circ. Res.* 99 1084–1091. 10.1161/01.RES.0000250046.69918.d5 17038640

[B162] Nikolaev V. O. Moshkov A. Lyon A. R. Miragoli M. Novak P. Paur H. (2010). Beta2-adrenergic receptor redistribution in heart failure changes cAMP compartmentation. *Science* 327 1653–1657. 10.1126/science.1185988 20185685

[B163] Oestreich E. A. Malik S. Goonasekera S. A. Blaxall B. C. Kelley G. G. Dirksen R. T. (2009). Epac and phospholipase C𝜖 regulate Ca2^+^ release in the heart by activation of protein kinase C𝜖 and calcium-calmodulin kinase II. *J. Biol. Chem.* 284 1514–1522. 10.1074/jbc.M806994200 18957419 PMC2615515

[B164] Orini M. Nanda A. Yates M. Di Salvo C. Roberts N. Lambiase P. D. (2017). Mechano-electrical feedback in the clinical setting: current perspectives. *Prog. Biophys. Mol. Biol.* 130 365–375. 10.1016/j.pbiomolbio.2017.06.001 28587763

[B165] Pereira L. Bare D. J. Galice S. Shannon T. R. Bers D. M. (2017). β-Adrenergic induced SR Ca2^+^ leak is mediated by an Epac-NOS pathway. *J. Mol. Cell. Cardiol.* 108 8–16. 10.1016/j.yjmcc.2017.04.005 28476660 PMC5523849

[B166] Pereira L. Métrich M. Fernández-Velasco M. Lucas A. Leroy J. Perrier R. (2007). The cAMP binding protein Epac modulates Ca2^+^ sparks by a Ca2^+^/calmodulin kinase signalling pathway in rat cardiac myocytes. *J. Physiol.* 583 685–694. 10.1113/jphysiol.2007.133066 17599964 PMC2277038

[B167] Petroff M. G. Kim S. H. Pepe S. Dessy C. Marbán E. Balligand J. L. (2001). Endogenous nitric oxide mechanisms mediate the stretch dependence of Ca2^+^ release in cardiomyocytes. *Nat. Cell Biol.* 3 867–873. 10.1038/ncb1001-867 11584267

[B168] Ponikowski P. Voors A. A. Anker S. D. Bueno H. Cleland J. G. Coats A. J. (2016). 2016 ESC Guidelines for the diagnosis and treatment of acute and chronic heart failure: the Task Force for the diagnosis and treatment of acute and chronic heart failure of the European Society of Cardiology (ESC) Developed with the special contribution of the Heart Failure Association (HFA) of the ESC. *Eur. Heart J.* 37 2129–2200. 10.1093/eurheartj/ehw128 27206819

[B169] Port J. D. Bristow M. R. (2001). Altered beta-adrenergic receptor gene regulation and signaling in chronic heart failure. *J. Mol. Cell. Cardiol.* 33 887–905. 10.1006/jmcc.2001.1358 11343413

[B170] Porter B. Bishop M. J. Claridge S. Behar J. Sieniewicz B. J. Webb J. (2017). Autonomic modulation in patients with heart failure increases beat-to-beat variability of ventricular action potential duration. *Front. Physiol.* 8:328 10.3389/fphys.2017.00328 PMC5447044 28611676

[B171] Priori S. G. Corr P. B. (1990). Mechanisms underlying early and delayed afterdepolarizations induced by catecholamines. *Am. J. Physiol.* 258 H1796–H1805. 10.1152/ajpheart.1990.258.6.H1796 2163219

[B172] Priori S. G. Mantica M. Napolitano C. Schwartz P. J. (1990). Early afterdepolarizations induced in vivo by reperfusion of ischemic myocardium. A possible mechanism for reperfusion arrhythmias. *Circulation* 81 1911–1920. 10.1161/01.CIR.81.6.1911 2344683

[B173] Priori S. G. Mantica M. Schwartz P. J. (1988). Delayed afterdepolarizations elicited in vivo by left stellate ganglion stimulation. *Circulation* 78 178–185. 10.1161/01.CIR.78.1.178 3383403

[B174] Priori S. G. Napolitano C. Memmi M. Colombi B. Drago F. Gasparini M. (2002). Clinical and molecular characterization of patients with catecholaminergic polymorphic ventricular tachycardia. *Circulation* 106 69–74. 10.1161/01.CIR.0000020013.73106.D8 12093772

[B175] Prosser B. L. Ward C. W. Lederer W. J. (2011). X-ROS signaling: rapid mechano-chemo transduction in heart. *Science* 333 1440–1445. 10.1126/science.1202768 21903813

[B176] Purohit A. Rokita A. G. Guan X. Chen B. Koval O. M. Voigt N. (2013). Oxidized Ca2^+^/calmodulin-dependent protein kinase II triggers atrial fibrillation. *Circulation* 128 1748–1757. 10.1161/CIRCULATIONAHA.113.003313 24030498 PMC3876034

[B177] Rakhit A. Maguire C. T. Wakimoto H. Gehrmann J. Li G. K. Kelly R. A. (2001). In vivo electrophysiologic studies in endothelial nitric oxide synthase (eNOS)-deficient mice. *J. Cardiovasc. Electrophysiol.* 12 1295–1301. 10.1046/j.1540-8167.2001.01295.x 11761419

[B178] Ravens U. (2003). Mechano-electric feedback and arrhythmias. *Prog. Biophys. Mol. Biol.* 82 255–266. 10.1016/S0079-6107(03)00026-9 12732284

[B179] Sag C. M. Santos C. X. Shah A. M. (2014). Redox regulation of cardiac hypertrophy. *J. Mol. Cell. Cardiol.* 73 103–111. 10.1016/j.yjmcc.2014.02.002 24530760

[B180] Sag C. M. Wadsack D. P. Khabbazzadeh S. Abesser M. Grefe C. Neumann K. (2009). Calcium/calmodulin-dependent protein kinase II contributes to cardiac arrhythmogenesis in heart failure. *Circ. Heart Fail.* 2 664–675. 10.1161/CIRCHEARTFAILURE.109.865279 19919992 PMC2835502

[B181] Sag C. M. Wagner S. Maier L. S. (2013). Role of oxidants on calcium and sodium movement in healthy and diseased cardiac myocytes. *Free Radic. Biol. Med.* 63 338–349. 10.1016/j.freeradbiomed.2013.05.035 23732518

[B182] Sagawa K. Lie R. K. Schaefer J. (1990). Translation of Otto frank’s paper “Die Grundform des arteriellen Pulses” zeitschrift für biologie 37: 483–526 (1899). *J. Mol. Cell. Cardiol.* 22 253–254. 10.1016/0022-2828(90)91459-K 2192068

[B183] Saini A. Kannabhiran M. Reddy P. Gopinathannair R. Olshansky B. Dominic P. (2016). Cardiac resynchronization therapy may be antiarrhythmic particularly in responders: a systematic review and meta-analysis. *JACC Clin. Electrophysiol.* 2 307–316. 10.1016/j.jacep.2015.10.007 29766889

[B184] Sánchez G. Pedrozo Z. Domenech R. J. Hidalgo C. Donoso P. (2005). Tachycardia increases NADPH oxidase activity and RyR2 S-glutathionylation in ventricular muscle. *J. Mol. Cell. Cardiol.* 39 982–991. 10.1016/j.yjmcc.2005.08.010 16242147

[B185] Sanchez-Alonso J. L. Bhargava A. O’Hara T. Glukhov A. V. Schobesberger S. Bhogal N. (2016). Microdomain-specific modulation of L-type calcium channels leads to triggered ventricular arrhythmia in heart failurenovelty and significance. *Circ. Res.* 119 944–955. 10.1161/CIRCRESAHA.116.308698 27572487 PMC5045818

[B186] Santangeli P. Rame J. E. Birati E. Y. Marchlinski F. E. (2017). Management of ventricular arrhythmias in patients with advanced heart failure. *J. Am. Coll. Cardiol.* 69 1842–1860. 10.1016/j.jacc.2017.01.047 28385314

[B187] Sato D. Xie L.-H. Sovari A. A. Tran D. X. Morita N. Xie F. (2009). Synchronization of chaotic early afterdepolarizations in the genesis of cardiac arrhythmias. *Proc. Natl. Acad. Sci. U.S.A.* 106 2983–2988. 10.1073/pnas.0809148106 19218447 PMC2651322

[B188] Saxon L. A. Bristow M. R. Boehmer J. Krueger S. Kass D. A. De Marco T. (2006). Predictors of sudden cardiac death and appropriate shock in the comparison of medical therapy, pacing, and defibrillation in heart failure (COMPANION) trial. *Circulation* 114 2766–2772. 10.1161/CIRCULATIONAHA.106.642892 17159063

[B189] Schillinger W. Fiolet J. W. Schlotthauer K. Hasenfuss G. (2003). Relevance of Na^+^–Ca2^+^ exchange in heart failure. *Cardiovasc. Res.* 57 921–933. 10.1016/S0008-6363(02)00826-X 12650870

[B190] Schobesberger S. Wright P. Tokar S. Bhargava A. Mansfield C. Glukhov A. V. (2017). T-tubule remodelling disturbs localized β2-adrenergic signalling in rat ventricular myocytes during the progression of heart failure. *Cardiovasc. Res.* 113 770–782. 10.1093/cvr/cvx074 28505272 PMC5437368

[B191] Schönleitner P. Schotten U. Antoons G. (2017). Mechanosensitivity of microdomain calcium signalling in the heart. *Prog. Biophys. Mol. Biol.* 130 288–301. 10.1016/j.pbiomolbio.2017.06.013 28648626

[B192] Schröder F. Handrock R. Beuckelmann D. J. Hirt S. Hullin R. Priebe L. (1998). Increased availability and open probability of single L-type calcium channels from failing compared with nonfailing human ventricle. *Circulation* 98 969–976. 10.1161/01.CIR.98.10.969 9737516

[B193] Scriven D. R. Moore E. D. (2013). Ca2^+^ channel and Na^+^/Ca2^+^ exchange localization in cardiac myocytes. *J. Mol. Cell. Cardiol.* 58 22–31. 10.1016/j.yjmcc.2012.11.022 23220152

[B194] Seo K. Rainer P. P. Lee D.-I. Hao S. Bedja D. Birnbaumer L. (2014). Hyperactive adverse mechanical stress responses in dystrophic heart are coupled to transient receptor potential canonical 6 and blocked by cGMP-protein kinase G modulation. *Circ. Res.* 114 823–832. 10.1161/CIRCRESAHA.114.302614 24449818 PMC3963144

[B195] Sham J. S. (1997). Ca2^+^ release-induced inactivation of Ca2^+^ current in rat ventricular myocytes: evidence for local Ca2^+^ signalling. *J. Physiol.* 500(Pt 2), 285–295. 9147317 10.1113/jphysiol.1997.sp022020 PMC1159383

[B196] Sham J. S. Cleemann L. Morad M. (1995). Functional coupling of Ca2^+^ channels and ryanodine receptors in cardiac myocytes. *Proc. Natl. Acad. Sci. U.S.A.* 92 121–125. 10.1073/pnas.92.1.121 7816800 PMC42829

[B197] Sharma V. K. Ramesh V. Franzini-Armstrong C. Sheu S.-S. (2000). Transport of Ca2^+^ from sarcoplasmic reticulum to mitochondria in rat ventricular myocytes. *J. Bioenerg. Biomembr.* 32 97–104. 10.1023/A:1005520714221 11768767

[B198] Shugg T. Johnson D. E. Shao M. Lai X. Witzmann F. Cummins T. R. (2018). Calcium/calmodulin-dependent protein kinase II regulation of IKs during sustained β-adrenergic receptor stimulation. *Heart Rhythm* 15 895–904. 10.1016/j.hrthm.2018.01.024 29410121 PMC5984714

[B199] Simon J. N. Duglan D. Casadei B. Carnicer R. (2014). Nitric oxide synthase regulation of cardiac excitation-contraction coupling in health and disease. *J. Mol. Cell. Cardiol.* 73 80–91. 10.1016/j.yjmcc.2014.03.004 24631761

[B200] Sipido K. R. Callewaert G. Carmeliet E. (1995). Inhibition and rapid recovery of Ca2^+^ current during Ca2^+^ release from sarcoplasmic reticulum in guinea pig ventricular myocytes. *Circ. Res.* 76 102–109. 10.1161/01.RES.76.1.102 8001267

[B201] Sipido K. R. Stankovicova T. Vanhaecke J. Flameng W. Verdonckc F. (1998). A critical role for L-type Ca2^+^ current in the regulation of Ca2^+^ release from the sarcoplasmic reticulum in human ventricular myocytes from dilated cardiomyopathy. *Ann. N. Y. Acad. Sci.* 853 353–356. 10.1111/j.1749-6632.1998.tb08298.x 10603978

[B202] Sipido K. R. Volders P. G. A. Vos M. A. Verdonck F. (2002). Altered Na/Ca exchange activity in cardiac hypertrophy and heart failure: a new target for therapy? *Cardiovasc. Res.* 53 782–805. 10.1016/S0008-6363(01)00470-9 11922890

[B203] Song L.-S. Sobie E. A. McCulle S. Lederer W. J. Balke C. W. Cheng H. (2006). Orphaned ryanodine receptors in the failing heart. *Proc. Natl. Acad. Sci. U.S.A.* 103 4305–4310. 10.1073/pnas.0509324103 16537526 PMC1449688

[B204] Song Y. Shryock J. C. Wagner S. Maier L. S. Belardinelli L. (2006). Blocking late sodium current reduces hydrogen peroxide-induced arrhythmogenic activity and contractile dysfunction. *J. Pharmacol. Exp. Ther.* 318 214–222. 10.1124/jpet.106.101832 16565163

[B205] Song Y.-H. Cho H. Ryu S.-Y. Yoon J.-Y. Park S.-H. Noh C.-I. (2010). L-type Ca2^+^ channel facilitation mediated by H_2_O_2_-induced activation of CaMKII in rat ventricular myocytes. *J. Mol. Cell. Cardiol.* 48 773–780. 10.1016/j.yjmcc.2009.10.020 19883656

[B206] Sossalla S. Fluschnik N. Schotola H. Ort K. R. Neef S. Schulte T. (2010). Inhibition of elevated Ca2^+^/calmodulin-dependent protein kinase II improves contractility in human failing myocardium. *Circ. Res.* 107 1150–1161. 10.1161/CIRCRESAHA.110.220418 20814023

[B207] Sukharev S. I. Blount P. Martinac B. Blattner F. R. Kung C. (1994). A large-conductance mechanosensitive channel in *E. coli* encoded by mscL alone. *Nature* 368 265–268. 10.1038/368265a0 7511799

[B208] Swaminathan P. D. Purohit A. Hund T. J. Anderson M. E. (2012). Calmodulin-dependent protein kinase II: linking heart failure and arrhythmias. *Circ. Res.* 110 1661–1677. 10.1161/CIRCRESAHA.111.243956 22679140 PMC3789535

[B209] Tavi P. Laine M. Weckström M. Ruskoaho H. (2001). Cardiac mechanotransduction: from sensing to disease and treatment. *Trends Pharmacol. Sci.* 22 254–260. 10.1016/S0165-6147(00)01679-5 11339977

[B210] Terracciano C. M. Hardy J. Birks E. J. Khaghani A. Banner N. R. Yacoub M. H. (2004). Clinical recovery from end-stage heart failure using left-ventricular assist device and pharmacological therapy correlates with increased sarcoplasmic reticulum calcium content but not with regression of cellular hypertrophy. *Circulation* 109 2263–2265. 10.1161/01.CIR.0000129233.51320.92 15136495

[B211] Tomaselli G. F. Marbán E. (1999). Electrophysiological remodeling in hypertrophy and heart failure. *Cardiovasc. Res.* 42 270–283. 10.1016/S0008-6363(99)00017-6 10533566

[B212] Toschi-Dias E. Rondon M. U. P. B. Cogliati C. Paolocci N. Tobaldini E. Montano N. (2017). Contribution of autonomic reflexes to the hyperadrenergic state in heart failure. *Front. Neurosci.* 11:162. 10.3389/fnins.2017.00162 28424575 PMC5372354

[B213] Tsuji Y. Opthof T. Kamiya K. Yasui K. Liu W. Lu Z. (2000). Pacing-induced heart failure causes a reduction of delayed rectifier potassium currents along with decreases in calcium and transient outward currents in rabbit ventricle. *Cardiovasc. Res.* 48 300–309. 10.1016/S0008-6363(00)00180-2 11054476

[B214] Ungerer M. Böhm M. Elce J. S. Erdmann E. Lohse M. J. (1993). Altered expression of beta-adrenergic receptor kinase and beta 1-adrenergic receptors in the failing human heart. *Circulation* 87 454–463. 10.1161/01.CIR.87.2.454 8381058

[B215] van Oort R. J. McCauley M. D. Dixit S. S. Pereira L. Yang Y. Respress J. L. (2010). Ryanodine receptor phosphorylation by CaMKII promotes life-threatening ventricular arrhythmias in mice with heart failure. *Circulation* 122 2669–2679. 10.1161/CIRCULATIONAHA.110.982298 21098440 PMC3075419

[B216] Varró A. Baláti B. Iost N. Takács J. Virág L. Lathrop D. A. (2000). The role of the delayed rectifier component IKs in dog ventricular muscle and Purkinje fibre repolarization. *J. Physiol.* 523 67–81. 10.1111/j.1469-7793.2000.00067.x 10675203 PMC2269783

[B217] Vegh A. Szekeres L. Parratt J. (1992). Preconditioning of the ischaemic myocardium; involvement of the L-arginine nitric oxide pathway. *Br. J. Pharmacol.* 107 648–652. 10.1111/j.1476-5381.1992.tb14501.x 1472963 PMC1907772

[B218] Veldkamp M. W. van Ginneken A. C. Opthof T. Bouman L. N. (1995). Delayed rectifier channels in human ventricular myocytes. *Circulation* 92 3497–3504. 10.1161/01.CIR.92.12.3497 8521572

[B219] Veldkamp M. W. Verkerk A. O. van Ginneken A. C. G. Baartscheer A. Schumacher C. de Jonge N. (2001). Norepinephrine induces action potential prolongation and early afterdepolarizations in ventricular myocytes isolated from human end-stage failing hearts. *Eur. Heart J.* 22 955–963. 10.1053/euhj.2000.2499 11428819

[B220] Venetucci L. A. Trafford A. W. O’Neill S. C. Eisner D. A. (2008). The sarcoplasmic reticulum and arrhythmogenic calcium release. *Cardiovasc. Res.* 77 285–292. 10.1093/cvr/cvm009 18006483

[B221] Verkerk A. O. Veldkamp M. W. Baartscheer A. Schumacher C. A. Klöpping C. van Ginneken A. C. G. (2001). Ionic mechanism of delayed afterdepolarizations in ventricular cells isolated from human end-stage failing hearts. *Circulation* 104 2728–2733. 10.1161/hc4701.099577 11723027

[B222] Vermeulen J. T. Mcguire M. A. Opthof T. Coronel R. de Bakker J. M. Klöpping C. (1994). Triggered activity and automaticity in ventricular trabeculae of failing human and rabbit hearts. *Cardiovasc. Res.* 28 1547–1554. 10.1093/cvr/28.10.1547 8001044

[B223] Viatchenko-Karpinski S. Kornyeyev D. El-Bizri N. Budas G. Fan P. Jiang Z. (2014). Intracellular Na^+^ overload causes oxidation of CaMKII and leads to Ca2^+^ mishandling in isolated ventricular myocytes. *J. Mol. Cell. Cardiol.* 76 247–256. 10.1016/j.yjmcc.2014.09.009 25252177 PMC4250389

[B224] Vielma A. Z. León L. Fernández I. C. González D. R. Boric M. P. (2016). Nitric oxide synthase 1 modulates basal and β-adrenergic-stimulated contractility by rapid and reversible redox-dependent S-nitrosylation of the heart. *PLoS One* 11:e0160813. 10.1371/journal.pone.0160813 27529477 PMC4986959

[B225] Volders P. G. Kulcśar A. Vos M. A. Sipido K. R. Wellens H. J. Lazzara R. (1997). Similarities between early and delayed afterdepolarizations induced by isoproterenol in canine ventricular myocytes. *Cardiovasc. Res.* 34 348–359. 10.1016/S0008-6363(96)00270-2 9205549

[B226] Volders P. G. Stengl M. van Opstal J. M. Gerlach U. Spätjens R. L. Beekman J. D. (2003). Probing the contribution of IKs to canine ventricular repolarization: key role for beta-adrenergic receptor stimulation. *Circulation* 107 2753–2760. 10.1161/01.CIR.0000068344.54010.B3 12756150

[B227] Volders P. G. Vos M. A. Szabo B. Sipido K. R. de Groot S. H. Gorgels A. P. (2000). Progress in the understanding of cardiac early afterdepolarizations and torsades de pointes: time to revise current concepts. *Cardiovasc. Res.* 46 376–392. 10.1016/S0008-6363(00)00022-5 10912449

[B228] von Anrep G. (1912). On the part played by the suprarenals in the normal vascular reactions of the body. *J. Physiol.* 45 307–317. 10.1113/jphysiol.1912.sp001553 16993158 PMC1512890

[B229] Waagstein F. Bristow M. R. Swedberg K. Camerini F. Fowler M. B. Silver M. A. (1993). Beneficial effects of metoprolol in idiopathic dilated cardiomyopathy. Metoprolol in Dilated Cardiomyopathy (MDC) Trial Study Group. *Lancet* 342 1441–1446. 10.1016/0140-6736(93)92930-R 7902479

[B230] Wagner S. Dybkova N. Rasenack E. C. L. Jacobshagen C. Fabritz L. Kirchhof P. (2006). Ca2^+^/calmodulin-dependent protein kinase II regulates cardiac Na^+^ channels. *J. Clin. Invest.* 116 3127–3138. 10.1172/JCI26620 17124532 PMC1654201

[B231] Wagner S. Rokita A. G. Anderson M. E. Maier L. S. (2013). Redox regulation of sodium and calcium handling. *Antioxid. Redox Signal.* 18 1063–1077. 10.1089/ars.2012.4818 22900788 PMC3567778

[B232] Wagner S. Ruff H. M. Weber S. L. Bellmann S. Sowa T. Schulte T. (2011). Reactive oxygen species-activated Ca/calmodulin kinase IIδ is required for late I(Na) augmentation leading to cellular Na and Ca overload. *Circ. Res.* 108 555–565. 10.1161/CIRCRESAHA.110.221911 21252154 PMC3065330

[B233] Wang H. Kohr M. J. Wheeler D. G. Ziolo M. T. (2008). Endothelial nitric oxide synthase decreases beta-adrenergic responsiveness via inhibition of the L-type Ca2^+^ current. *Am. J. Physiol. Heart Circ. Physiol.* 294 H1473–H1480. 10.1152/ajpheart.01249.2007 18203845 PMC2744450

[B234] Wang H. Viatchenko-Karpinski S. Sun J. Györke I. Benkusky N. A. Kohr M. J. (2010). Regulation of myocyte contraction via neuronal nitric oxide synthase: role of ryanodine receptor S-nitrosylation. *J. Physiol.* 588 2905–2917. 10.1113/jphysiol.2010.192617 20530114 PMC2956906

[B235] Wang J. Ma Y. Sachs F. Li J. Suchyna T. M. (2016). GsMTx4-D is a cardioprotectant against myocardial infarction during ischemia and reperfusion. *J. Mol. Cell. Cardiol.* 98 83–94. 10.1016/j.yjmcc.2016.07.005 27423272 PMC5026603

[B236] Wang W. Zucker I. H. (1996). Cardiac sympathetic afferent reflex in dogs with congestive heart failure. *Am. J. Physiol.* 271 R751–R756. 10.1152/ajpregu.1996.271.3.R751 8853400

[B237] Wehrens X. H. Lehnart S. E. Reiken S. R. Marks A. R. (2004). Ca2^+^ /calmodulin-dependent protein kinase II phosphorylation regulates the cardiac ryanodine receptor. *Circ. Res.* 94 e61–e70. 10.1161/01.RES.0000125626.33738.E2 15016728

[B238] Wei X.-H. Yu S.-D. Ren L. Huang S.-H. Yang Q.-M. Wang P. (2017). Inhibition of late sodium current suppresses calcium-related ventricular arrhythmias by reducing the phosphorylation of CaMK-II and sodium channel expressions. *Sci. Rep.* 7:981. 10.1038/s41598-017-01056-0 28428622 PMC5430524

[B239] Westenbrink B. D. Edwards A. G. McCulloch A. D. Brown J. H. (2013). The promise of CaMKII inhibition for heart disease: preventing heart failure and arrhythmias. *Expert Opin. Ther. Targets* 17 889–903. 10.1517/14728222.2013.809064 23789646 PMC5551677

[B240] White E. (2006). Mechanosensitive channels: therapeutic targets in the myocardium? *Curr. Pharm. Des.* 12 3645–3663. 17073665 10.2174/138161206778522083

[B241] Wit A. L. Rosen M. R. (1983). Pathophysiologic mechanisms of cardiac arrhythmias. *Am. Heart J.* 106 798–811. 10.1016/0002-8703(83)90003-0 6310978

[B242] Wright P. T. Nikolaev V. O. O’Hara T. Diakonov I. Bhargava A. Tokar S. (2014). Caveolin-3 regulates compartmentation of cardiomyocyte beta2-adrenergic receptor-mediated cAMP signaling. *J. Mol. Cell. Cardiol.* 67 38–48. 10.1016/j.yjmcc.2013.12.003 24345421 PMC4266930

[B243] Xiao B. Sutherland C. Walsh M. P. Chen S. R. W. (2004). Protein kinase A phosphorylation at serine-2808 of the cardiac Ca2^+^-release channel (ryanodine receptor) does not dissociate 12.6-kDa FK506-binding protein (FKBP12.6). *Circ. Res.* 94 487–495. 10.1161/01.RES.0000115945.89741.22 14715536

[B244] Xie L.-H. Chen F. Karagueuzian H. S. Weiss J. N. (2009). Oxidative-stress-induced afterdepolarizations and calmodulin kinase II signaling. *Circ. Res.* 104 79–86. 10.1161/CIRCRESAHA.108.183475 19038865 PMC2747806

[B245] Xie Y. Sato D. Garfinkel A. Qu Z. Weiss J. N. (2010). So little source, so much sink: requirements for afterdepolarizations to propagate in tissue. *Biophys. J.* 99 1408–1415. 10.1016/j.bpj.2010.06.042 20816052 PMC2931729

[B246] Xu K. Y. Huso D. L. Dawson T. M. Bredt D. S. Becker L. C. (1999). Nitric oxide synthase in cardiac sarcoplasmic reticulum. *Proc. Natl. Acad. Sci. U.S.A.* 96 657–662. 10.1073/pnas.96.2.657 9892689 PMC15192

[B247] Xu L. Eu J. P. Meissner G. Stamler J. S. (1998). Activation of the cardiac calcium release channel (ryanodine receptor) by poly-S-nitrosylation. *Science* 279 234–237. 10.1126/science.279.5348.234 9422697

[B248] Yamada K. A. Corr P. B. (1992). Effects of β-adrenergic receptor activation on intracellular calcium and membrane potential in adult cardiac myocytes. *J. Cardiovasc. Electrophysiol.* 3 209–224. 10.1111/j.1540-8167.1992.tb00968.x

[B249] Yamaguchi Y. Iribe G. Nishida M. Naruse K. (2017). Role of TRPC3 and TRPC6 channels in the myocardial response to stretch: linking physiology and pathophysiology. *Prog. Biophys. Mol. Biol.* 130 264–272. 10.1016/j.pbiomolbio.2017.06.010 28645743

[B250] Yan Y. Liu J. Wei C. Li K. Xie W. Wang Y. (2008). Bidirectional regulation of Ca2^+^ sparks by mitochondria-derived reactive oxygen species in cardiac myocytes. *Cardiovasc. Res.* 77 432–441. 10.1093/cvr/cvm047 18006452

[B251] Zahradníková A. Minarovic I. Venema R. C. Mészáros L. G. (1997). Inactivation of the cardiac ryanodine receptor calcium release channel by nitric oxide. *Cell Calcium* 22 447–454. 10.1016/S0143-4160(97)90072-5 9502194

[B252] Zeng J. Rudy Y. (1995). Early afterdepolarizations in cardiac myocytes: mechanism and rate dependence. *Biophys. J.* 68 949–964. 10.1016/S0006-3495(95)80271-7 7538806 PMC1281819

[B253] Zhang H. Gomez A. M. Wang X. Yan Y. Zheng M. Cheng H. (2013). ROS regulation of microdomain Ca2^+^ signalling at the dyads. *Cardiovasc. Res.* 98 248–258. 10.1093/cvr/cvt050 23455546

[B254] Zhang M. Perino A. Ghigo A. Hirsch E. Shah A. M. (2013). NADPH oxidases in heart failure: poachers or gamekeepers? *Antioxid. Redox Signal.* 18 1024–1041. 10.1089/ars.2012.4550 22747566 PMC3567780

[B255] Zima A. V. Blatter L. A. (2006). Redox regulation of cardiac calcium channels and transporters. *Cardiovasc. Res.* 71 310–321. 10.1016/j.cardiores.2006.02.019 16581043

[B256] Zygmunt A. C. Goodrow R. J. Weigel C. M. (1998). INaCa and ICl(Ca) contribute to isoproterenol-induced delayed after depolarizations in midmyocardial cells. *Am. J. Physiol.* 275 H1979–H1992. 9843796 10.1152/ajpheart.1998.275.6.H1979

